# Construction and application of a multifunctional CHO cell platform utilizing Cre/*lox* and Dre/*rox* site-specific recombination systems

**DOI:** 10.3389/fbioe.2023.1320841

**Published:** 2023-12-20

**Authors:** Chen Zhang, Feng Chang, Hui Miao, Yunhui Fu, Xikui Tong, Yu Feng, Wenyun Zheng, Xingyuan Ma

**Affiliations:** ^1^ State Key Laboratory of Bioreactor Engineering, School of Biotechnology, East China University of Science and Technology, Shanghai, China; ^2^ Shanghai Key Laboratory of New Drug Design, School of Pharmacy, East China University of Science and Technology, Shanghai, China

**Keywords:** CHO, Cre/lox, Dre/*rox*, CRISPR/Cas9, site-specific recombination, hotspot, production stability

## Abstract

During the development of traditional Chinese hamster ovary (CHO) cell lines, target genes randomly integrate into the genome upon entering the nucleus, resulting in unpredictable productivity of cell clones. The characterization and screening of high-yielding cell lines is a time-consuming and expensive process. Site-specific integration is recognized as an effective approach for overcoming random integration and improving production stability. We have designed a multifunctional expression cassette, called CDbox, which can be manipulated by the site-specific recombination systems Cre/*lox* and Dre/*rox.* The CDbox expression cassette was inserted at the *Hipp11*(*H11*) locus hotspot in the CHO-K1 genome using CRISPR/Cas9 technology, and a compliant CHO-CDbox cell platform was screened and obtained. The CHO-CDbox cell platform was transformed into a pool of EGFP-expressing cells using Cre/*lox* recombinase-mediated cassette exchange (RMCE) in only 2 weeks, and this expression remained stable for at least 75 generations without the need for drug stress. Subsequently, we used the Dre/*rox* system to directly eliminate the *EGFP* gene. In addition, two practical applications of the CHO-CDbox cell platform were presented. The first was the quick construction of the Pembrolizumab antibody stable expression strain, while the second was a protocol for the integration of surface-displayed and secreted antibodies on CHO cells. The previous research on site-specific integration of CHO cells has always focused on the single functionality of insertion of target genes. This newly developed CHO cell platform is expected to offer expanded applicability for protein production and gene function studies.

## 1 Introduction

Chinese hamster ovary (CHO) cells have long been the cell line of choice for therapeutic recombinant protein expression, and approximately 70% of all globally approved therapeutic recombinant proteins have been produced in CHO cell lines (Lalonde and Durocher, 2017; Dhara et al., 2018; Dahodwala and Lee, 2019). Over the past 2 decades, with the development of optimized media, process control, and cell engineering, mammalian cell production has made qualitative leaps, with CHO protein production exceeding 10 g/L by batch replenishment (Kelley, 2009; Nguyen and Zimmer, 2023). However, longer cultivation times, lower product yield, and phenotypic heterogeneity of transgenic cell clones have been identified as limitations of mammalian cell lines compared with *E. coli,* yeast, and insect cell lines (Dalton and Barton, 2014; Li et al., 2016; Wells and Robinson, 2017). In order to meet the growing market demand for biopharmaceuticals, continuous innovation in manufacturing processes is required to achieve higher volume productivity in a shorter period, while ensuring stable product quality and reducing production costs. This is a key research topic in the biopharmaceutical industry (Sarah et al., 2016; Sharker and Rahman, 2020; Bryan et al., 2021).

Traditional CHO cell line generation has heavily relied on randomized integration and large-scale screening of highly productive cell lines (Yusufi et al., 2017). Despite significant advances in production processes and hardware equipment over this period, the basic approach has remained relatively unchanged (Bandaranayake and Almo, 2014). Thus, subclone groups are selected from cell pools based on their productivity and other characteristics, such as cell viability, glycosylation, and stability (Lai et al., 2013). The entire development process usually takes 6–12 months, which is time, labor, and cost intensive (Shirahata, 2000). The transcriptional activity of a gene locus is controlled by its chromatin state and genetic instability, and sites in the genome that are abnormally stable and have high transcriptional activity are called “hotspots” by researchers (Thomas et al., 1986; Koduri et al., 2001). Many efforts have been made to identify transcriptional hotspots in host genomes and dozens of CHO hotspots have been reported, including *Fer1L4, Rosa26, Ywhae, Hprt, Hipp11 and C12orf35* (Gaidukov et al., 2018; Hamaker and Lee, 2018; Zhao et al., 2018; Chi et al., 2019; Fan et al., 2020; Kim et al., 2021). The *Hipp11* (*H11*) locus is a non-coding sequence situated between the conserved *DRG1* and *EIF4ENIF1* genes (Hippenmeyer et al., 2010). It does not include the introns or exons of the encoded genes and enables gene expression without interrupting the functional genes. The *H11* locus has been extensively researched in mammalian cells, including mice, pigs, human embryonic stem cells, and induced pluripotent stem cells (Tasic et al., 2011; Zhu et al., 2014; Ruan et al., 2015; Browning et al., 2019). The studies have shown that it has the potential for stable transgene integration and high-level expression. A previous study indicated that the *H11* locus in CHO-S cells is a “safe harbor” genomic pattern for stable and efficient transgene knock-in and expression (Chi et al., 2019).

To avoid the variability of randomly integrating transgenes and to shorten the overall development time for cell lines, a site-specific recombinase technique has been developed to incorporate target genes into pre-characterized chromosomal motifs (Baer and Bode, 2001; Olorunniji et al., 2016). Site-specific recombinases recognize and bind to their respective recognition sites to perform precise insertions, deletions, or inversions of DNA fragments (Brown et al., 2011; Rutherford and Van Duyne, 2014). We refer to double swaps consisting of site-specific recombinase targeting sites as recombinase-mediated cassette exchange (RMCE) (Zhang et al., 2015; Srirangan et al., 2020). With gene exchange by the RMCE method, DNA breaks and rejoins occur only between the corresponding loci, resulting in protein-producing cell lines stably expressed at fixed loci (Kameyama et al., 2010). Many site-specific recombinase systems have been attempted to mediate expression cassette exchange by site-specific recombination in mammalian cells, e.g., Cre/*lox*, Flp/*FRT*, BxB1 recombinase, and PhiC31 integrase (Zhang et al., 2015; Inniss et al., 2017; Pourtabatabaei et al., 2021; Min et al., 2022). Among the studied and employed recombinases, Cre recombinase stands out as one of the most intensively investigated ones, exhibiting higher site specificity and efficiency (Turan et al., 2013). It is widely considered as the top site-specific recombinase for genome engineering (Kameyama et al., 2010). Genentech established a Cre-based RMCE platform by screening for transcriptionally active sites in the CHO genome and screened it to obtain stable cell lines expressing monoclonal antibodies with a quantitative productivity of 3–4 pg/cell/day (Crawford et al., 2013).

Earlier attempts to apply RMCE to productive mammalian cells have been difficult due to the need to first generate recombinase recognition sites (also known as landing pads) as recombinase targets in the cellular genome (Qiao et al., 2009; Kameyama et al., 2010). With the development of gene editing technology and the discovery of hotspots, a combinatorial method for nuclease homology-mediated targeted integration of RMCE into genomic hotspots has been developed (Dangi et al., 2018; Kalkan et al., 2023). This method avoids the drawbacks of stochastic integration and allows faster generation of productive mammalian cell lines (Phan et al., 2017). In this study, we developed a multifunctional CDbox expression cassette and utilized CRISPR/Cas9 technology to insert it into the *H11* locus, resulting in the creation of a CHO-CDbox cell platform. The Cre/*lox*-mediated RMCE effect and the deletion of the Dre/*rox* system were validated in the CHO-CDbox cell platform using an *EGFP* reporter gene donor. Additionally, we have provided an example of quickly constructing Pembrolizumab antibody expression lines on CHO-CDbox platform cells using only the Cre/*lox* system; and we have demonstrated a protocol that integrates the display and secretion of antibody libraries on the surface of CHO cells through the combined action of the Cre/*lox* and Dre/*rox* systems.

## 2 Materials and methods

### 2.1 sgRNA design and PX458-sgRNA plasmid construction

The *H11* locus was defined as the sequence between the conserved genes *DRG1* and *EIF4ENIF1*, according to the CHO-K1 (2014 version) gene annotation, *DRG1* Gene ID: 100764489 (84466-104272), *EIF4ENIF1* Gene ID: 100762835 (39687-79338), the *H11* sequence ranges from 79339 to 84465, covering 5,127 base pairs. The sgRNAs were generated based on the gene sequence of *H11* by optimizing CRISPR design through online tools (http://crispor.tefor.net/), and the three highest scoring and lowest off-target effects were selected as sgRNA1, sgRNA2, and sgRNA3. The target sequences of sgRNAs were synthesized as shown in Supplementary Table S1.

The upstream and downstream primers of sgRNA in Supplementary Table S1 were mixed with 1 μL each, mixed with 7 μL of ddH_2_O and 1 μL of 10 × PCR buffer, denatured in a PCR instrument at 95°C for 5 min, and then removed and cooled naturally to room temperature to obtain the complexed sgRNA double-stranded DNA. Plasmid pX458 (Addgene plasmid #48138) was digested with BbsI (NEB) at 37°C for 3 h and recovered using a gel purification kit (Tiangen). sgRNA double-stranded DNA was ligated to the pX458 linearized plasmid with T4 ligase (TAKARA) and transformed into DH5α competent cells. The pX458-sgRNA plasmid was extracted by Plasmid mini-extraction kit (Tiangen) after incubation with ampicillin antibiotics.

### 2.2 Donor vector construction

CDbox liner donor construction: CMV enhancer, *mCherry* sequence, and BGH poly(A) were amplified from the existing plasmid pCDNA3.1(+)-mCherry; *T2A-PuroR* sequence was from plasmid pX459 (Addgene plasmid # 62988); the homology arm of *H11* depends on the final choice of sgRNA; *loxP* sequence is 5′-ATA​ACT​TCG​TAT​AGC​ATA​CAT​TAT​ACG​AAG​TTA​T-3'; *lox2272* sequence is 5′- ATA​ACT​TCG​TAT​AGG​ATA​CTT​TAT​ACG​AAG​TTA​T-3'; *roxP* sequence is 5′-TAA​CTT​TAA​ATA​ATG​CCA​ATT​ATT​TAA​AGT​TA-3'.

pMV-HygR-EGFP donor construction: *EGFP* sequence was obtained from plasmid pCDNA3.1(+)-EGFP kept in the laboratory; Hygromycin B resistance gene (*HygR*) sequence was obtained from plasmid pLV-hTERT-IRES-Hygro (Addgene plasmid # 85140); and pMV-*loxP*-*lox2272* plasmid was the gene synthesis plasmid.

pMV-HC-LC-HygR donor construct: Pembrolizumab full-length antibody sequence was derived from Pembrolizumab plasmid (Addgene plasmid #85434).

pMV-VNAR-Fc-TM-HygR donor construction: the *VNAR* antibody library sequence was obtained from a synthetic phage library kept in the laboratory; the *Fc* fragment of the heavy chain antibody was obtained from the Pembrolizumab plasmid (Plasmid #85434); the TM was platelet-derived growth factor receptor (PDGFR) transmembrane domain from the synthetic plasmid pMV-*roxP*-TM; and the *T2A-HygR* sequence was from the previously constructed plasmid pMV-HC-LC-HygR.

The specific construction method and procedure are shown in Supplementary Figure S1, Supplementary Figure S2, Supplementary Figure S3, and Supplementary Figure S4, and the primers used are shown in Supplementary Table S2.

### 2.3 Cell culture, transfection, stable cell line generation, and clonal selection

CHO-K1 cells (ATCC: CCL-61) were cultured using Ham’s F-12 medium (Gibco) supplemented with 10% fetal bovine serum and 1% penicillin-streptomycin-amphotericin (Solarbio) in a cell culture incubator at 37°C in a humid environment with 5% CO_2_. Cells with 80%–90% confluence were digested with 0.25% trypsin (Solarbio) and cultured in passages at a 1:4 ratio, changing the medium every 2 days.

One day before cell transfection, the target cells were digested and spread to 6-well plates, and about 5×10^5^ cells were added evenly to each well, and the medium was replaced with fresh medium the next day. The transfection mix was added sequentially in 100 μL opti-MEM media (Gibco) with 2 μg total DNA (including plasmid and donor), 4 μL Lipo8000 Transfection Reagent (Beyotime), and mixed gently. The transfection mixture was added drop by drop to the 6-well plate gently shaken crosswise to mix well, and incubated in a CO_2_ incubator. The transfection experiment was repeated three times. The fluorescence rate of the cells was monitored by inverted fluorescence microscopy (Olympus) and flow cytometry (BD FACScan) at different time intervals according to the specific experimental requirements.

After 72 h or 96 h of transfection, antibiotics were added to the culture medium for screening. The medium was changed every 2–3 days until the emergence and amplification of stable clones. Antibiotic concentrations were used for screening the stably integrated cell lines: puromycin concentration of 10 μg/mL and hygromycin concentration of 200 μg/mL.

Individual clones from the cell pool were acquired through limited dilution. Cells digested with trypsin were diluted in culture medium and inoculated at the density of 0.5 cells per well of the 96-well plates. Following approximately 10 days of incubation, the cells in the well of single-cell clones were transferred to 24-well plates and then expanded into 6-well plates or T25 culture flasks for further analysis.

### 2.4 5'/3′junction PCR amplification

The genomes of the cells were extracted with the Genome Extraction Kit (Tiangen), and 5′/3′Junction PCR was performed using 2 × Premix DNA Polymerase (TAKARA), with upstream and downstream primers designed to be on the outside of the 5′/3′homology arm and inside the expression cassette, respectively. The primer positions are shown schematically in Supplementary Figure S5A; Supplementary Figure S7A. The primer sequences used are shown in Supplementary Table S3.

### 2.5 Cell proliferation experiment

Cell proliferation experiments were performed using the xCELLigence RTCA Instrument (Agilent). 2000 target cells were counted by plate counting, diluted with 200 μL of medium, added to the E-Plate 16, and operated according to the RTCA Instrument manufacturer’s instructions, which automatically recorded the cell growth trend. The RTCA Instrument automatically records cell growth trends.

### 2.6 Flow cytometry analysis of cell fluorescence

5 × 10^5^ target cells were digested and collected into 1.5 mL centrifuge tubes, washed twice with PBS, resuspended in 200 μL PBS, and analyzed for fluorescence rate using the flow cytometry. The channel of detection was selected according to the needs of the experiment: FITC (EGFP) or Y610 (mCherry).

For antibody display analysis experiments, 1 × 10^6^ washed target cells were resuspended with 1 mL PBS, 1 μL FITC-conjugated Goat Anti-Human IgG (H + L) (Proteintech) was added under dark conditions, incubated for 1 h at 4°C, and washed twice with pre-cooled PBS before being resuspended with 200 μL PBS. The cells were analyzed by flow cytometry for the percentage of antibody presentation.

### 2.7 qPCR for gene copy number detection

Plasmid pCDNA3.1 (+)-CDbox with known copy number was used as a standard and diluted to 10^7^, 10^6^, 10^5^, 10^4^, 10^3^, 10^2^, and 10 copies/μL according to the gradient of plasmid concentration, and used as a DNA template for the standard curve to perform qPCR amplification using a CFX96 fluorescence quantitative PCR instrument (Bio-Rad). The gene copy number was calculated by the formula:

$$Copies/\mu L=\frac{6.02\times 10^{23}\times C\times 10^{-9}}{Length\times 660}$$

where C is the concentration of the plasmid (ng/μL) and Length is the size of the plasmid sequence (bp).

In this experiment, the upstream primer is 5′-CCC​ACA​ACG​AGG​ACT​ACA​CAC​C-3′ and the downstream primer is 5′-GGG​CTT​GTA​CTC​GGG​TCA​TTG-3'. The reaction system was 2 × qPCR SYBR Green Master Mix (Yeasen) 10 μL, upstream and downstream primers (5 μM) 1 μL each, template 1 μL, add H_2_O to make up to 20 μL; amplification conditions for 95°C 5 min; 40 ×: 95°C 10 s, 60°C 30 s. The standard curve was plotted with the log copy number value of the standards as the horizontal coordinate and the measured Ct value as the vertical coordinate, and the amplification efficiency was calculated according to the standard curve. The genome of the cells (2.6 GB) was extracted, and the absolute copy number of the target gene in the sample was calculated by bringing in the Ct value of the cell genome sample.

### 2.8 ELISA

The ELISA plate was coated with 100 μL of 2 mg/mL PD-1 antigen (DIMA BIOTECH) per well and incubated at 4°C overnight. After blocking non-specific binding with 1% BSA for 1 h, the plates were washed with PBST, and successive concentration gradients of Pembrolizumab standards (DIMA BIOTECH) and diluted sample antibodies were added to the corresponding wells and incubated for 1 h. After washing with PBST, the plates were incubated for 1 h with the addition of HRP-conjugated Goat Anti-Human IgG (H + L) (Proteintech) was incubated at room temperature for 30 min then TMB substrate solution (Solarbio) was added and the absorbance at 405 nm was detected using a microplate reader (Thermo Scientific).

### 2.9 Western blot

2 × 10^5^ target cells were diluted with 2 mL of medium, inoculated in 6-well plates, and cultured for 2 days. The culture supernatant was collected after centrifugation at 12,000 g for 10 min and then concentrated 4-fold using a 3KD ultrafiltration centrifuge tube (Millipore). Cultured cells were lysed with 200 μL of RIPA lysis buffer (Solarbio) containing 1 mM PMSF on ice, and the lysed supernatant was collected by centrifugation at 12,000 g for 10 min at 4°C and the total protein concentration was determined by BCA protein assay kit (Beyotime).

The culture medium supernatant samples and cell lysis samples were boiled for 10 min after adding loading buffer, separated by 12% SDS-PAGE gel, and transferred to 0.22 μm PVDF membrane (Millipore) after electrophoresis. After blocking with 5% skimmed milk powder for 1 h, the membrane was incubated with primary antibody at 4°C overnight and secondary antibodies at room temperature for 1 hour, and then visualized and photographed using an ECL chemiluminescence kit (Yeasen). In this study, the following antibodies were used: anti-beta actin antibody from mouse (Abcam), HRP-conjugated Goat Anti-Mouse IgG (H + L) (Proteintech), HRP-conjugated Goat Anti-Human IgG (H + L) (Proteintech).

## 3 Result

### 3.1 Integration of CDbox expression cassette into *H11* locus utilizing CRISPR/Cas9 technology

**FIGURE 1:**
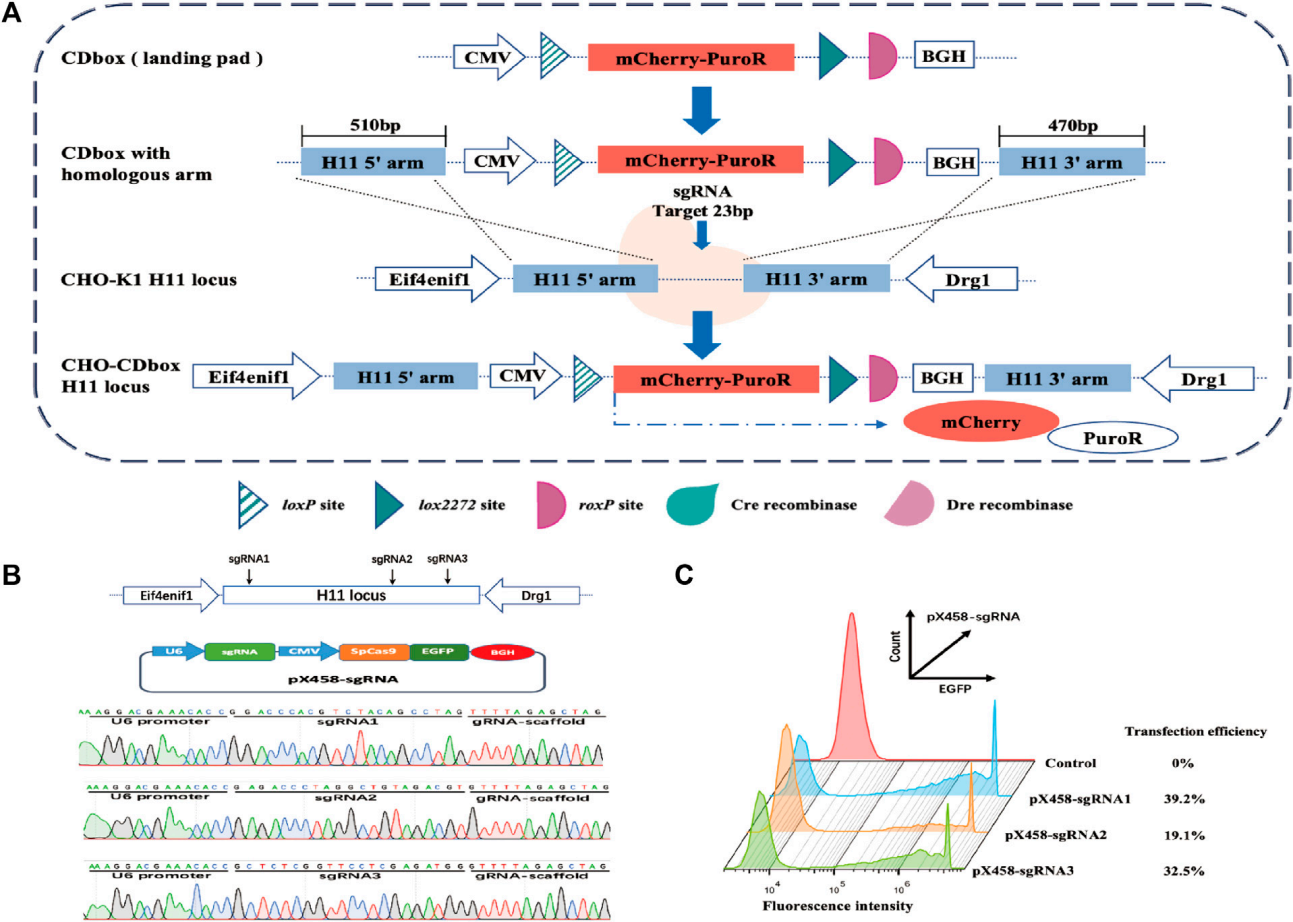
Targeted integration of CDbox expression cassette utilizing the CRISPR/Cas9 system. **(A)** Schematic diagram of CRISPR/Cas9-mediated site-specific integration of CDbox expression cassette. The CDbox expression cassette comprised a complete expression system including the CMV promoter, the gene of mCherry and PuroR fusion protein, and the BGH polyA. The expression system contained a Cre recombinase recognition site *loxP* before the start codon, a Cre recombinase recognition site *lox2272,* and a Dre recombinase recognition site *roxP* after the stop codon. The ends of the CDbox were linked to the homology arms of the target site and inserted into the *H11* locus by CRISPR/Cas9 technology-mediated homologous recombination, and the CHO-CDbox cell line was obtained by screening. **(B)** Location of sgRNA targeting the *H11* locus and sequence map of the construct into the pX458-sgRNA vector. **(C)** The transfection efficiency of CHO-K1 cells transfected with the pX458-sgRNA plasmid by flow cytometry.

We developed a CDbox expression cassette corresponding to the Cre/*lox* and Dre/*rox* systems. It has a complete expression system comprising the CMV promoter, mCherry and PuroR fusion protein, and BGH polyA. There is a Cre recombinase recognition site *loxP* upstream of the fusion protein start codon. Downstream of the stop codon, there are *lox2272* and *roxP* sites, which are recognized by the Cre and the Dre recombinase, respectively (Figure 1A).

The gene sequences of the *H11* locus were evaluated using the online tool website of Feng Zhang’s laboratory (http://crispor.tefor.net/), and then the sequence was screened to identify the three sgRNAs, sgRNA1, sgRNA2, and sgRNA3, with the highest scores and lowest off-target effects, which are shown in Supplementary Table S1. The sgRNA oligonucleotides were synthesized and recombined with pX458 plasmids, resulting in pX458-sgRNA1, pX458-sgRNA2, and pX458-sgRNA3 (Figure 1B). After transfecting each vector separately into CHO-K1 cells, flow analysis revealed transfection efficiencies of 39.2%, 19.1%, and 32.5%, respectively (Figure 1C). Since pX458-sgRNA1 showed the highest transfection efficiency, we used its targeted cleavage site position as the insertion site for the gene. The 510 bp long sequence at the 5′end and the 470 bp long sequence at the 3′end of the insertion site were incorporated into both ends of the CDbox as homology arm sequences, and then the CDbox donor sequence targeting the *H11* locus was obtained (Supplementary Figure S1; Supplementary Figure S1A).

**FIGURE 2:**
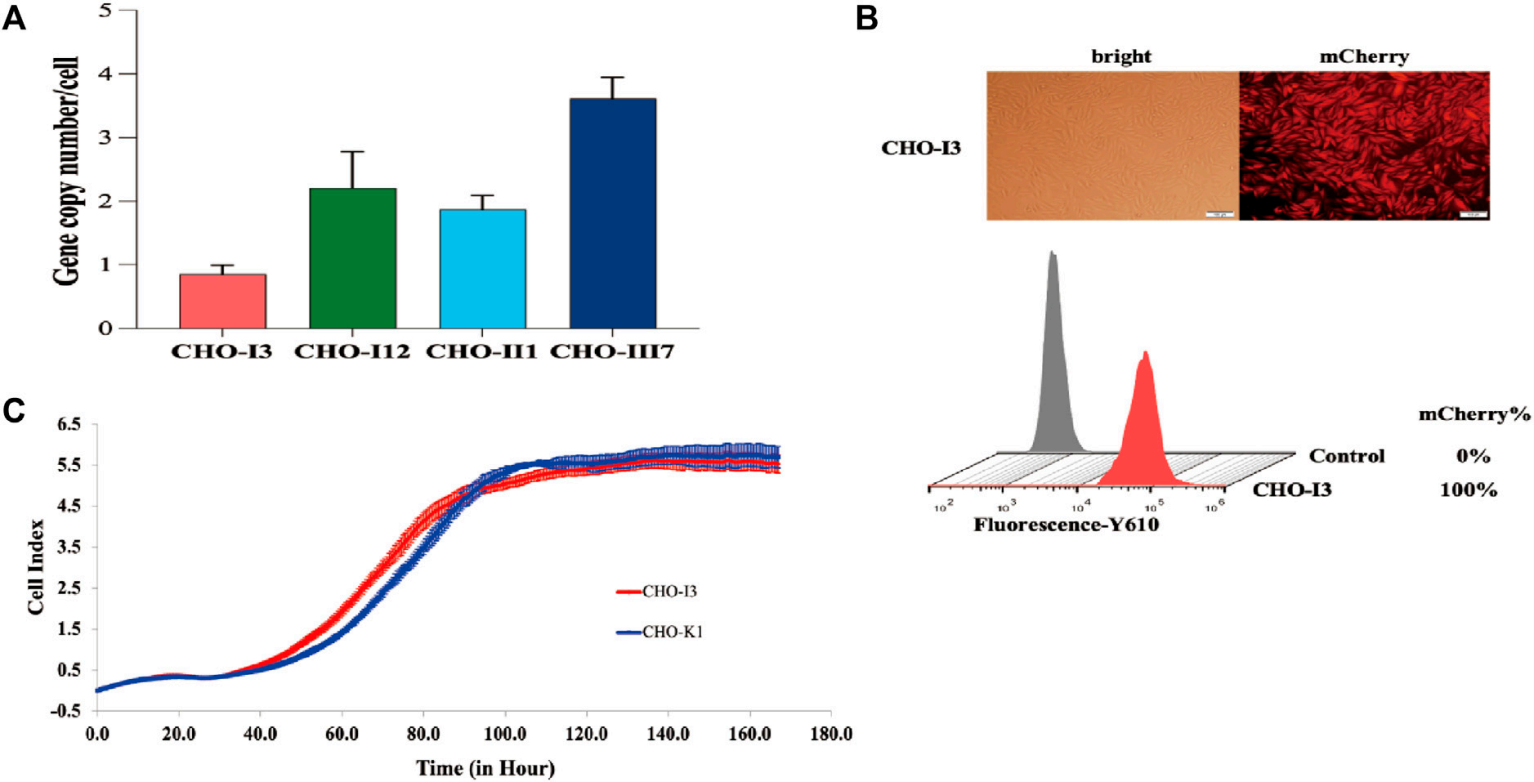
Performance test of CHO-I3 cell line. **(A)** Examination of the copy number of CDbox in the genome of candidate cell strains. Absolute quantification of copy number by qPCR revealed that only the CHO-I3 cell line was a single copy (mean ± S.D., n = 3 independent experiments). **(B)** Morphology of CHO-I3 cells under an inverted fluorescence microscope and fluorescence rate detected by flow cytometry (scale bar, 100 μm) **(C)** Comparison of the proliferative status of the CHO-I3 cell line with that of the wild strain of CHO-K1 by utilizing the RTCA Instrument. 2000 cells were spread in E-Plate 16 for 7 days for cell proliferation experiments (3 replicate wells).

The CDbox donor sequence and the pX458-sgRNA1 plasmid were co-transfected into CHO-K1 cells. After 72 h, the medium containing puromycin was replaced, and a pool of puromycin-resistant cells was obtained following a 10-day screening process. The cell pool underwent monoclonal cell culture in a 96-well plate. Afterwards, 20 monoclonal cell strains with red fluorescence and robust growth were chosen for further expansion. The genomes of these selected cells were extracted and subjected to a 3′and 5′Junction PCR test (Supplementary Figure S5A). The correct 5′and 3′junction PCR bands were sequenced for 7 out of the 20 monoclonal cell strains (Supplementary Figure S5B). Only 4 monoclonal cell strains, CHO-I3, CHO-I12, CHO-II1, and CHO-III7, were found to be correctly sequenced, while the remaining 3 monoclonal cell strains had partial deletions and errors in the sequencing results. We analyzed the copy number of CDbox in each cell and found only one copy in the CHO-I3 genome. (Figure 2A). We believe that in the genomes of the other 3 cell lines, the CDbox expression cassette was integrated into multiple genomic loci in addition to the *H11* locus. The red fluorescence of the CHO-I3 cell strain exhibited a 100% intensity as determined through flow cytometry analysis (Figure 2B). We compared the proliferation viability of the CHO-I3 cell strain and wild-type CHO-K1 cell lines using RTCA Instrument. Our analysis revealed no significant difference in growth rate between the 2 cell lines (Figure 2C). Since the CHO-I3 cell strain carried a single copy of CDbox at the *H11* locus and its growth rate was not lower than that of wild-type cells, CHO-I3 was considered to meet the requirements of CHO-CDbox. Our next endeavor is to test its feasibility as a CHO-CDbox cell platform.

### 3.2 Feasibility of CHO-I3 cell strain as the CHO-CDbox cell platform

To assess the feasibility of CHO-I3 cell strain as a CHO-CDbox cell platform, we designed and constructed a donor vector “pMV-Hygro-EGFP” using EGFP protein as a reporter protein. The donor vector was combined with the *roxP* sequence between the *HygR* gene and the *EGFP* gene through PCR amplification and overlapping extension (Supplementary Figure S2; Supplementary Figure S3A). The *loxP* site and *lox2272* site sequences were situated before the start codon and after the stop codon, respectively.

Two methods for introducing Cre recombinase into cells were initially tested. The first method involved co-transfecting the donor vector and Cre recombinase expression plasmid pCDNA3.1-Cre into CHO-I3 cells using a 1:4 mass ratio. The second method was to add 5 μM of TAT-Cre recombinase (the N-terminal of the recombinase containing cell-penetrating peptide) to the medium 12 h after transfection of the donor vector into cells. Subsequently, green fluorescence rates were analyzed by flow cytometry at 48 h, 72 h, 96 h, 120 h, 144 h, 168 h, and 192 h after transfection. The rate of green fluorescence increases rapidly after transfection of pCDNA3.1-Cre into cells, reaching a maximum of 8% at 96 h (Supplementary Figure S6A). We estimated the RMCE efficiency of the Cre recombinase is 20%, based on liposomal transfection efficiency of 40%. The TAT-Cre recombinase was able to enter the cells through the use of cell-penetrating peptides and the fluorescence rate was found to peak at 120 h, slightly higher than the previous procedure.

**FIGURE 3:**
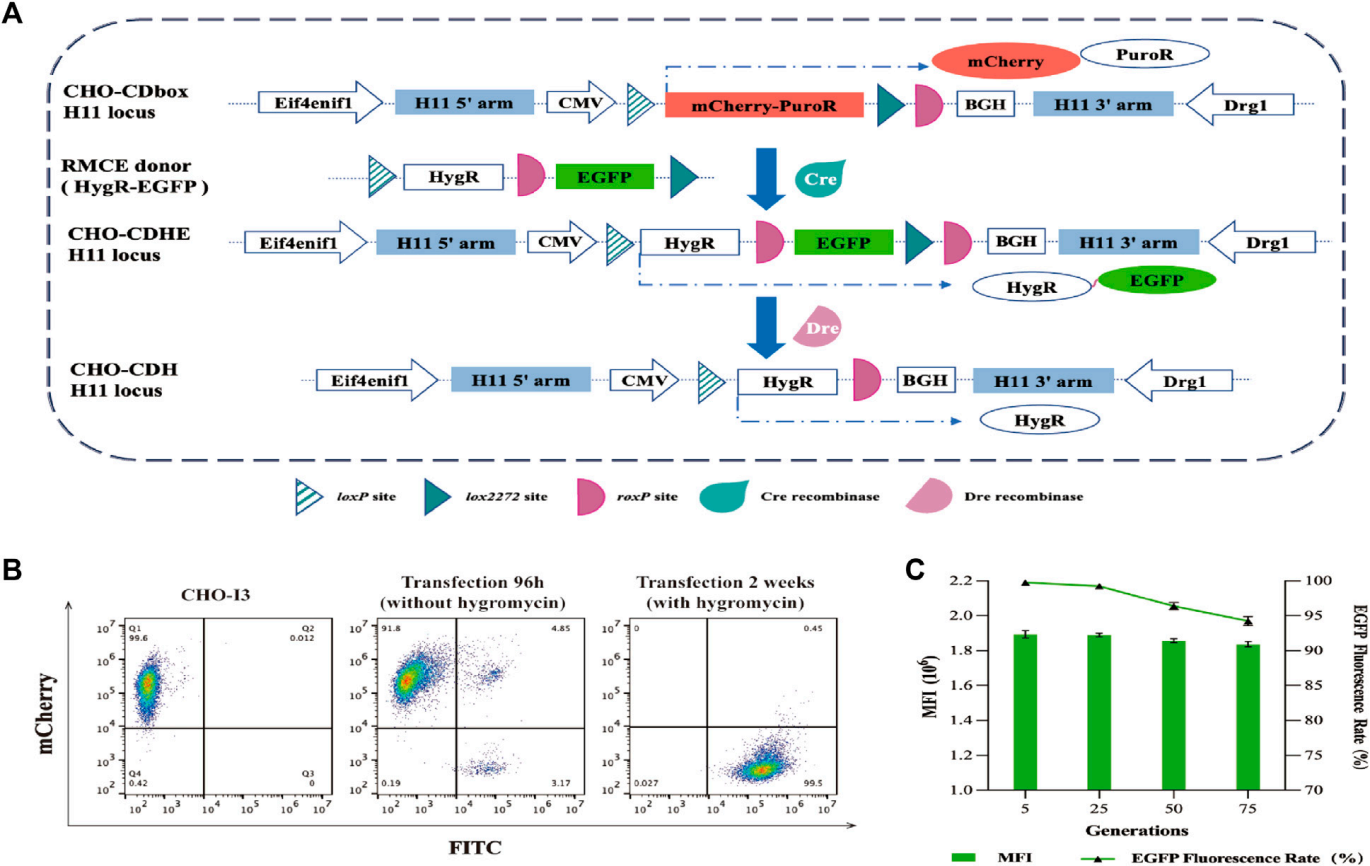
Mode of action of Cre/lox and Dre/*rox* in CHO-CDbox cells, and the flow cytometry analysis plot of fluorescence ratios of CHO-CDHE cell pools. **(A)** Schematic diagram of Cre/*lox*-based RMCE and Dre/*rox*-based deletion. By utilizing Cre recombinase-mediated RMCE, the mCherry-PuroR protein gene was replaced with the HygR-EGFP donor. Following this, the CHO-CDHE cell pool was acquired through hygromycin screening. Dre recombinase subsequently eliminated the EGFP tagged between two isotropic *roxP* sites in the CDbox of CHO-CDHE. **(B)** Flow cytometry results of the screening process of CHO-CDHE by Cre/*lox*-based RMCE. The figure illustrated the rate of green fluorescent expression cells before and after hygromycin screening. **(C)** Fluorescence expression rate and mean fluorescence intensity (MFI) of CHO-CDHE cells in different generations. The fluorescence expression rate and mean fluorescence intensity (MFI) of CHO-CDHE cells in the fifth, 25th, 50th, and 75th generations were measured by flow cytometry without antibiotics (mean ± S.D., *n* = 3 independent experiments).

The plasmid-based approach of Cre recombinase delivery into cells was chosen for its convenience and time-saving advantages. After 96 h of transfection, the medium was replaced with fresh medium containing 200 μg/mL of hygromycin for a 10-day screening culture to establish a pool of CHO-CDbox-HygR-EGFP (CHO-CDHE) cells. Flow cytometry analysis revealed a 99.5% green fluorescence rate of the cell pool screened by hygromycin (Figure 3B). Due to the possibility of undegraded mCherry protein affecting the result of flow analysis, over half of the green fluorescent cells were found to detect red fluorescence simultaneously, as evidenced by the flow analysis graphs before the transfection of 96 h along with antibiotics, despite the mCherry gene being replaced with the EGFP gene. The percentage of cells that co-expressed green fluorescent protein and red fluorescent protein was less than 0.5% after 10 days of exposure to antibiotics. Furthermore, no cells independently exhibiting red fluorescence were observed. It can be hypothesized that all the surviving cells were CHO-CDHE cells that had undergone RMCE.

To assess the stability of the CHO-CDHE cell pool, it was subjected to continuous passaging culture without the addition of hygromycin. The fluorescence expression rate and mean fluorescence intensity (MFI) were analyzed by sampling cells from the fifth, 25th, 50th, and 75th generations, calculated based on 1 day as one passaging. The results illustrate that the CHO-CDHE cell pool’s fluorescence expression rate was nearly 100% at the 5th and 25th generations, with a minor decline to 96.4% and 94.2% at the 50th and 75th generations, respectively. Additionally, the MFI of CHO-CDHE cells remained almost unchanged between the 5th and 75th generation as measured by flow cytometric statistics (Figure 3C). After 2.5 months of passaging (75 generations), 94.2% of the positive clones in the CHO-CDHE cell pool exhibited a highly stable expression of green fluorescence, confirming the transcriptional activity and expression stability of the *H11* locus following Cre-mediated RMCE.

**FIGURE 4:**
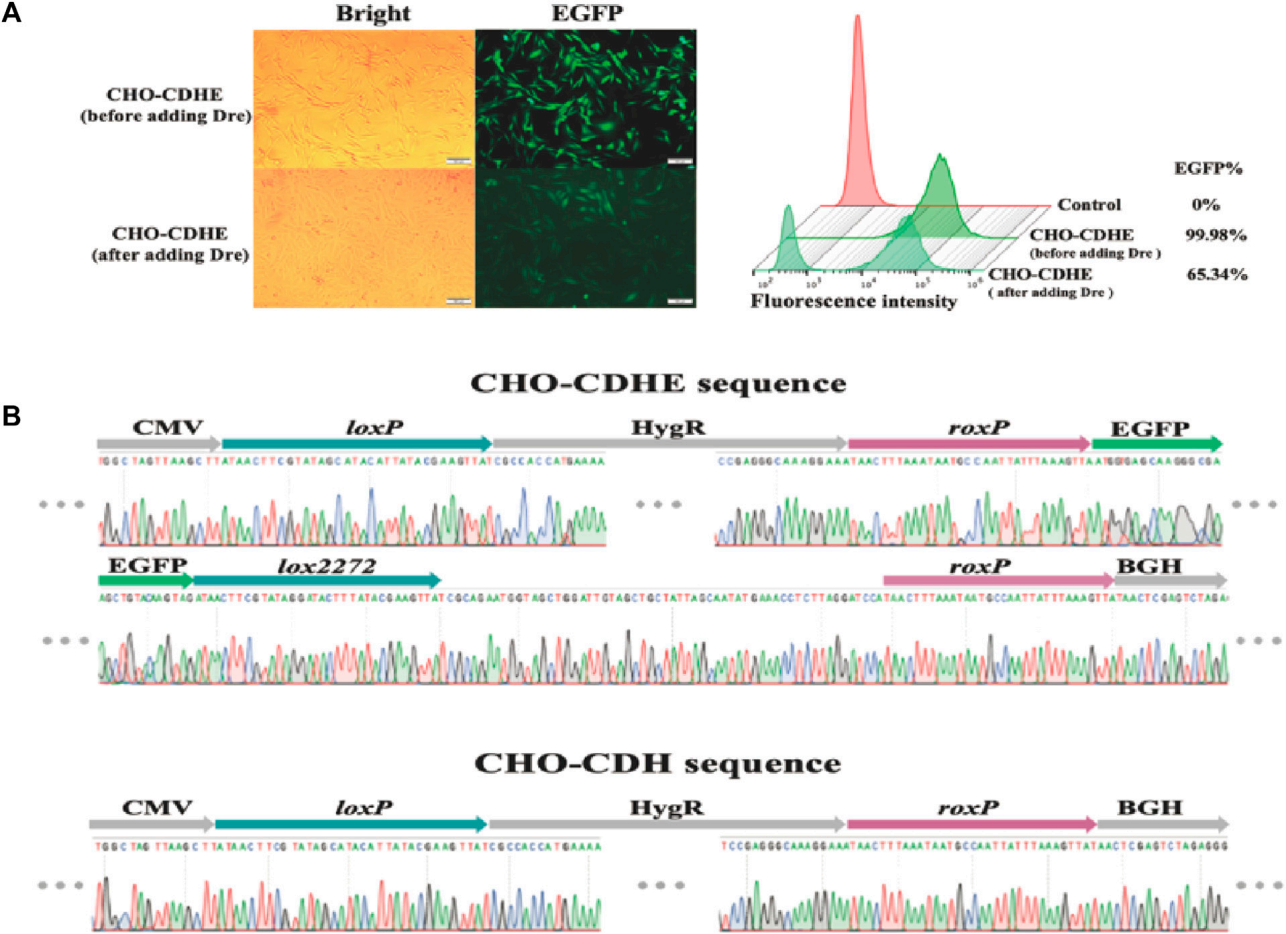
Changes of CHO-CDHE cell pool before and after Dre recombinase treatment. **(A)** Morphology of CHO-CDHE cells under an inverted fluorescence microscope (left) and fluorescence rate (right) detected by flow cytometry before and after Dre/*rox*-based deletion. After Dre recombinase’s activity, some cells in the CHO-CDbox cell pool lost fluorescence (scale bar, 100 μm). **(B)** Peak maps of key sequences at the *H11* locus in CHO-CDHE cells and CHO-CDH monoclonal cells. In the CHO-CDH cell, the *EGFP* gene between the two *roxP* sites was removed by Dre recombinase.

Subsequently, we assessed the impact of Dre recombinase on the CHO-CDHE cell pool. The effects of the two methods of Dre recombinase entry into cells were compared with the same approach as that used for Cre recombinase. The experimental results demonstrated that when Dre recombinase was introduced as a plasmid, the percentage of cells not expressing fluorescence remained under 20% over time (Supplementary Figure S6B). This result was limited by the transfection efficiency of the plasmid and the interference of undegraded EGFP. TAT-Dre recombinase caused a rapid reduction in green fluorescence rate. Despite the interference of undegraded EGFP, the rate decreased to near 60% after 168 h. Therefore, TAT-Dre recombinase incubation was employed to cleave the *roxP* sites. After 120 h of TAT-Dre recombinase treatment, we examined the cells using fluorescence microscopy and flow cytometry and found that approximately one-third of the cells did not show green fluorescence (Figure 4A). Eight monoclonal cell strains expressing green fluorescence (CHO-CDHE-1∼8) and eight monoclonal cell strains that did not express green fluorescence (CHO-CDH-1∼8) were isolated from the pool of cells treated with Dre recombinase. The genomes of these cell strains were extracted and the sequences of the CDbox were amplified by PCR to obtain sequencing data. It was determined that the sequencing results were consistent (Figure 4B). All the CHO-CDH cell strains did not express green fluorescence because Dre recombinase excised the *EGFP* gene between the two *roxP* sites.

After examining the results of the Cre-based RMCE and Dre recombinase cleavage gene, we determined that the CHO-I3 cell strain met the requirements for the CHO-CDbox cell platform. We subsequently employed the CHO-I3 cell strain as the CHO-CDbox cell platform for two example applications.

### 3.3 Rapid antibody production strain construction with CHO-CDbox cell platform

**FIGURE 5:**
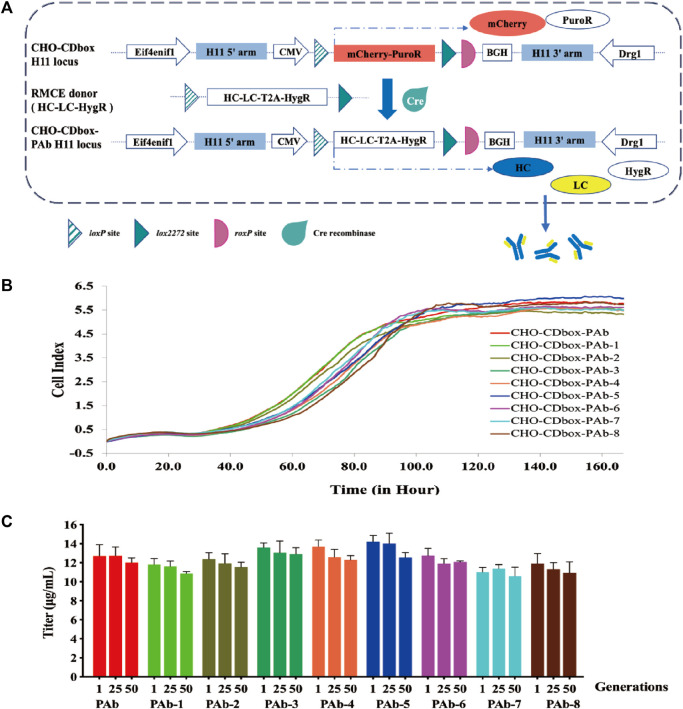
Performance evaluation of antibody expression strains constructed by CHO-CDbox cell platform. **(A)** Schematic diagram of construction antibody stable expression strain by Cre/*lox*-based RMCE. Utilizing Cre recombinase-mediated RMCE, the mCherry-PuroR protein gene was replaced with the HC-LC-HygR donor. Following this, the CHO-CDbox-PAb cell pool was acquired through hygromycin screening. **(B)** Comparison of proliferation curves of monoclonal antibody strains. A comparison of 7-day proliferation profiles of the CHO-CDbox-PAb cell pool and its eight monoclonal antibody strains was conducted through the RTCA Instrument. **(C)** Comparison of antibody secretion of monoclonal antibody strains at different generations. The Elisa assay was used to detect the antibody secretion of the CHO-CDbox-PAb cell pool and its 8 monoclonal antibody strains in the first, 25th, and 50th generations (mean ± S.D., n = 3 independent experiments).

CHO-CDbox cell platform was utilized for quickly constructing Pembrolizumab antibody strains. We designed and constructed a donor vector “pMV-HC-LC-HygR” (Figure 5A; Supplementary Figure S3). The donor was co-transfected with pCDNA3.1-Cre plasmid for 96 h. Subsequently, a 10-day hygromycin resistance screen was conducted to acquire CHO-CDbox-PAb, a cell pool for producing Pembrolizumab antibodies consistently. The CHO-CDbox-PAb cells pool obtained after 10 days of pressurization by hygromycin was subjected to flow cytometry, which showed that the percentage of dark cells was 98.42% and the mCherry fluorescence expression rate was 1.58% (Supplementary Figure S8). We guess that it is due to incomplete degradation of the mCherry protein in a fraction of the cells. The CHO-CDbox-PAb cells pool was resuscitated and cultured again, and the flow cytometry analyses of the first, fifth, 25th, and 50th generation cell pools showed that the percentage of dark cells was consistently close to 100%. Thus the CHO-CDbox-PAb cell pool we obtained remained genetically stable after 50 generations.

We randomly selected eight monoclonal cell strains (CHO-CDbox-PAb-1∼8) from the CHO-CDbox-PAb cell pool for culture. The genomes of the cell pool and the selected monoclonal cell strains underwent characterization via 5’/3’ Junction PCR amplification (Supplementary Figure S7A). No discernible differences were observed in the Junction PCR-amplified fragments of the eight monoclonal strains and the cell pool (Supplementary Figure S7B). The Junction PCR amplified fragments were subsequently recovered and sequenced, with identical sequencing results (results not shown). We performed qPCR of the Pembrolizumab antibody sequence on the CHO-CDbox-PAb cell pool and the CHO-CDbox-PAb-1∼8 monoclonal cell lines to evaluate their respective copy numbers, and the results showed that they were both single copy number (Supplementary Figure S7C). A comparison was conducted between the proliferation curves of the CHO-CDbox-PAb cell pool and the eight monoclones using the RTCA Instrument. The proliferation curves results revealed no significant differences in the rate and tendency of cell proliferation between the cell pool and the eight monoclonal cell strains (Figure 5B).

The CHO-CDbox-PAb cell pool and its eight monoclonal cell strains were cultured with the hygromycin-free medium. 1×10^6^ cells each from the first, 25th, and 50th generations were collected, diluted with 2 mL of medium and inoculated in 6-well plates. The culture supernatant was collected 72 h after incubation, and its antibody secretion was analyzed using ELISA. Overall, the variation in antibody secretion between monoclonal cell strains and cell pools was minimal (Figure 5C). The results showed that within generation first, CHO-CDbox-PAb cell pool showed antibody secretion in the range of 11.43–12.93 μg/mL, while CHO-CDbox-PAb-5 showed the highest secretion at 13.91–14.86 μg/mL and CHO-CDbox-PAb-7 the lowest at 10.85–11.28 μg/mL. The difference in the production of antibodies among individual monoclonal cell strains was within 25%, with a maximum difference of 12% when compared to the cell pool. There was a slight decrease in antibody secretion for each monoclonal cell line in the 50th generation in comparison to the first generation. The CHO-CDbox-PAb cell pool experienced a reduction of 7.32% in secretion, whereas CHO-CDbox-PAb-6 presented the smallest scale-down of only 5.27%, while CHO-CDbox-PAb-4 exhibited the most significant drop in secretion, with an average of 13.87 μg/mL which declined to 12.07 μg/mL, indicating a reduction of 12.98%. Based on the results of this study, it was concluded that the antibody-stabilized CHO-CDbox-PAb cell pool screened using the CHO-CDbox cell platform exhibits small inter-individual variation with a high degree of homogeneity and expression stability.

### 3.4 Mammalian cell surface antibody display via CHO-CDbox system

We will provide an example of using the CHO-CDbox cell platform to combine cell display and antibody secretion. CHO-CDbox cell platform integrates a solitary copy of CDbox at the *H11* locus, ensuring that each mammalian cell-surface antibody display exhibits only one antibody. Once the displaying process is complete, Dre recombinase can transform the antibody-displaying cells into antibody-secreting cells.

**FIGURE 6:**
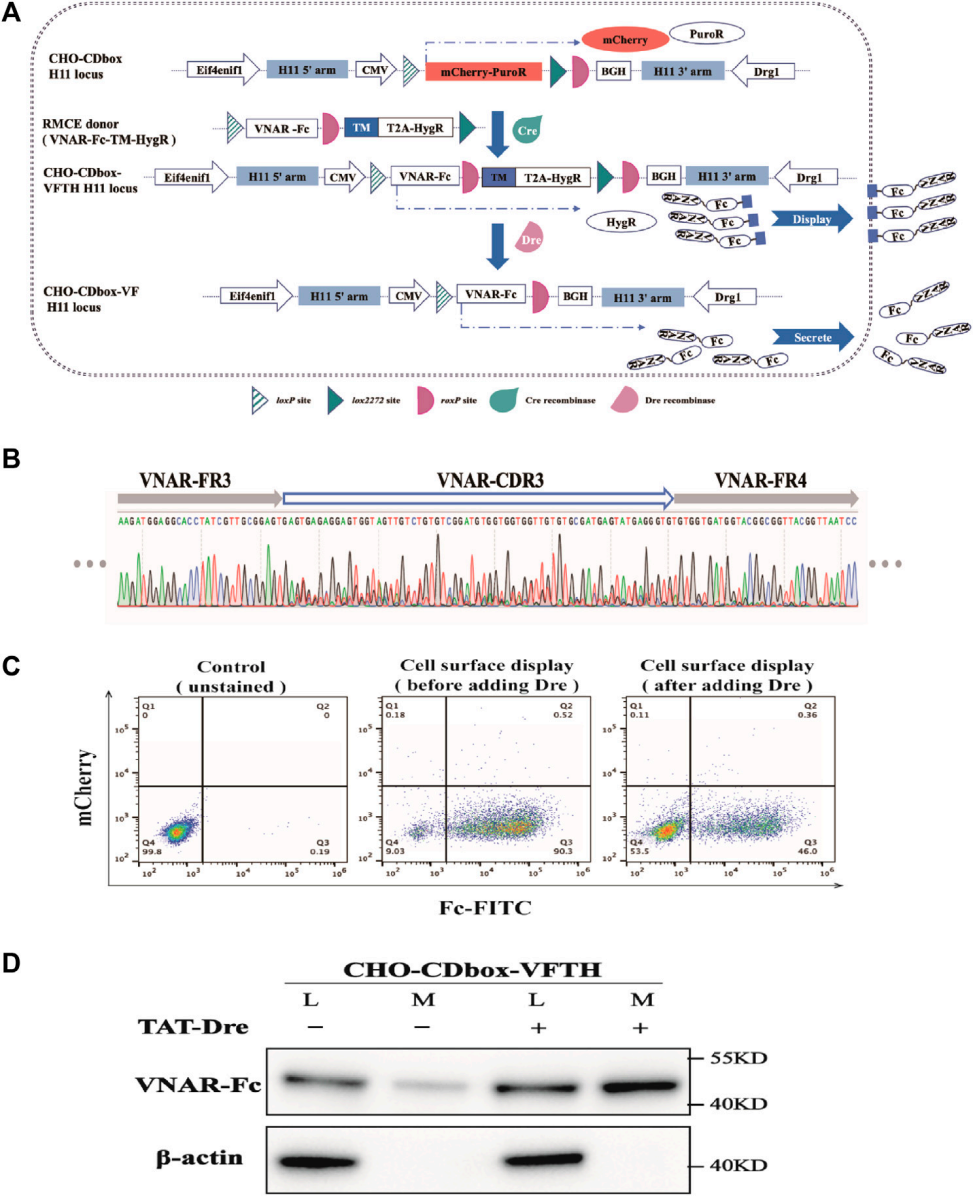
Experimental demonstration of the integration of CHO cell surface antibody display and expression using CHO-CDbox cell platform. **(A)** Schematic diagram of the principle of integration of antibody display and antibody secretion. Using Cre/*lox*-mediated RMCE, the VNAR-Fc-TM-HygR donor replaced the mCherry-PuroR protein gene. The CHO-CDbox-VFTH cell pool showcasing VNAR-Fc on the cell surface was acquired through hygromycin screening. Later, Dre recombinase removed the *TM-HygR* located between the two isotropic *roxP* sites in CHO-CDbox-VFTH, resulting in the extracellular secretion of VNAR-Fc. **(B)** Peak maps of CDR3 in VNAR from CHO-CDbox-VFTH cell pool. The CDR3 peak map appeared highly complex with overlapping peaks, suggesting the presence of the CHO-CDbox-VFTH cell pool as a library of antibodies. **(C)** Flow cytometry analysis plot of FITC flow antibody labeled CHO-CDbox-VFTH cell pools. The percentage of cells displaying VNAR-Fc antibody on the surface pre- and post-Dre recombinase action is illustrated through flow cytometry, utilizing FITC-coupled anti-human IgG (H + L) antibody to mark the Fc fragment on the cell surface. **(D)** Western blot analysis graph showing the level of VNAR-Fc secretion from CHO-CDbox-VFTH cell pool. VNAR-Fc levels in cell lysate and extracellular medium were assessed via Western blot before and after treatment with Dre recombinase. The addition of the Dre enzyme significantly increased the secretion of VNAR-Fc in the extracellular medium. L: cell lysate; M: extracellular medium.

We constructed the antibody library donor vector “pMV-VNAR-Fc-TM-HygR” by PCR amplification and overlapping extension (Figure 6A; Supplementary Figure S4). The VNAR was developed as a library of shark nanoantibodies by our laboratory members. The *VNAR*’s N-terminal has a signal peptide while its c-terminal was fused with the heavy chain Fc fragment of Pembrolizumab. This was followed by the *roxP* locus, the transmembrane domain sequence *TM* from Platelet-derived growth factor receptor (PDGFR), and the N-terminal *HygR* resistance gene with *T2A*. We co-transfected the donor vector and the pCDNA3.1-Cre plasmid into CHO-CDbox cells for 96 h, followed by screening in 10-day pressurized cultures with hygromycin to obtain the CHO-CDbox-VNAR-Fc-TM-HygR (CHO-CDbox-VFTH) antibody library cell pool. Transmembrane domain TM immobilized VNAR-Fc on the surface of CHO-CDbox-VFTH cells. Screening for target antibodies can be performed in formal experiments but was not performed for this example. We found that the CDR3 sequence of *VNAR* produced a significant overlapping peak when the genome of the CHO-CDbox-VFTH cell was extracted and sequenced (Figure 6B). This indicated that the cell pool group can be considered a cell library.

TAT-Dre recombinase was added into the cell pool medium to cleave the *TM* and *HygR* between *roxP* sites in the CHO-CDbox-VFTH cell genome. Without the TM, VNAR-Fc was secreted into the medium in response to the signaling peptide. As a result, CHO-CDbox-VFTH cells changed from a display antibody strain to a secreted antibody strain. After treating CHO-CDbox-VFTH cells with TAT-Dre recombinase for 144 h, the display antibody cells were labeled with FITC-conjugated goat anti-human IgG (H + L) and analyzed using flow cytometry. The percentage of cells that could display VNAR-Fc on the surface was significantly reduced, as indicated by the decrease in the fluorescence rate of cell labeling from 90.3% to 46.0% following TAT-Dre recombinase treatment (Figure 6C). We performed a Western blot test on equal amounts of cell culture supernatants before and after TAT-Dre recombinase treatment. Our results showed that the culture supernatants of the cell pools treated with TAT-Dre recombinase had pronounced antibody bands. In contrast, the samples taken before treatment had very shallow bands (Figure 6D). This suggests that after TAT-Dre recombinase treatment, a portion of CHO-CDbox-VFTH cells in the pool secreted VNAR-Fc into the supernatant. A small quantity of VNAR-Fc was also found in the culture supernatant concentrate of the cell pool before TAT-Dre treatment. This was thought to be the result of a small amount of *roxP* breaking naturally and the detachment of the antibody fragment from the transmembrane proteins during the display process. In formal experiments, antibodies can be collected directly from the supernatant of TAT-Dre recombinase-treated cells as the next step in the experiment, or antibody-secreting cell strains can be obtained directly by isolating monoclonal cells.

## 4 Discussion

The target gene is randomly integrated into the genome after nucleus entry, resulting in most cell clones having no fixed transgene integration sites and copy numbers (Oberbek et al., 2011; Hamaker and Lee, 2018). Since the insertion site of the target gene and the number of inserted copies of the gene are uncontrollable, the resulting randomly integrated cell lines may suffer from problems such as “positional effect” (Hamaker and Lee, 2018; Smith et al., 2020). As the number of culture generations increases, there may be a decline in production stability and lower levels of expression (Shin and Lee, 2020). The precise control of the insertion site for the target gene and maintenance of recombinant gene expression is crucial for engineering mammalian cells (Wirth et al., 2007). In this study, we chose the *H11* hotspot to create the CDbox cell platform. The CDbox expression cassette, containing genes for *mCherry* and *PuroR*, was incorporated into the *H11* locus utilizing CRISPR/Cas9 technology. We screened the CHO-I3 cell strain, which we verified through insertion site sequencing to be correct, had a single copy of the CDbox expression cassette, and had a value-added curve that did not significantly differ from CHO-K1. As a result, we chose it as the CHO-CDbox platform cell line. Next, we confirmed its ability to efficiently accomplish Cre/*lox*-based RMCE and Dre/*rox*-based cleavage using donor sequences with EGFP as a reporter protein. Afterward, we demonstrated the rapid construction of an antibody expression using only Cre recombinase and described an experimental protocol for integrated antibody display and secretion in CHO cells achieved through the combined pre- and post-recombination of Cre and Dre recombinases.

Here, we applied the Dre recombinase in CHO cells for the first time. The Dre recombinase, a recently developed recombinase, was discovered to be highly homologous to Cre recombinase, possessing a similar mechanism of action, recombination efficiency, and specificity (Brian and Jeffrey, 2004; Jensen et al., 2016; Madina et al., 2018). Tests on *E. coli,* HEK293 cells, zebrafish, mouse embryonic stem cells, and transgenic mouse lines expressing recombinant recombinases showed that Dre recombinases were an excellent candidate for combining with well-established recombinases Cre, Flp, and PhiC31 to generate combinatorial strategies due to its lack of cross-reactivity with them (Anastassiadis et al., 2009; Szilard et al., 2014; Chuang et al., 2015; Jin et al., 2021). In this report, we constructed a CHO-CDbox cell platform by the tandem combination of Cre/*lox* and Dre/*rox* systems. Using this cell platform, we can first greatly reduce the time required to construct stably expressing cell lines. Under the action of RMCE of Cre recombinase, we quickly obtained a CHO-CDHE cell pool with a fluorescence expression rate of up to 99.5% in 2 weeks. Secondly, since the donor “pMV-HygR-EGFP” used in our construction process did not contain a promoter, the cells could only survive and proliferate if they expressed the hygromycin resistance gene by precisely targeted exchange with the CDbox in the *H11* locus. It determined a high degree of homogeneity between individuals of the cell pool obtained by the RMCE system. Finally, after the exchange function of Cre recombinase was independently performed, Dre recombinase was used again to delete unnecessary genes. During the construction process, we examined the effects of Cre and Dre recombinases on intracellular gene editing through gene sequence sequencing, which verified that Cre and Dre recombinases had good cutting efficiency on the CHO cell genome, and there was no cross-reaction between Cre and Dre recombinases.

In industrial manufacturing, CHO can experience reduced yield during the later stages of production, so the stability of the integration site is one of the most important aspects (Pristovek et al., 2019). Previous studies have given us great confidence in the *H11* locus as a new potential CHO-K1 hotspot, and our experimental studies have demonstrated it. CHO-CDbox cell platform was constructed by inserting a CDbox liner donor into *H11* by CRISPR/Cas9 technology. In 20 monoclonal cells, 7 of them were found to have undergone site integration, with an efficiency of 35%. Despite further sequencing, only four monoclonal (20%) genome insertions were completely correct, indicating that the *H11* site can be efficiently integrated using CRISPR/Cas9 technology.

Using Cre-based RMCE, we obtained a CHO-CDHE cell pool expressing EGFP and a CHO-CDbox-PAb cell pool stably expressing Pembrolizumab full-length antibodies by 2 weeks. We examined the MFI and percentage of fluorescent cells in CHO-CDHE cell pools cultured to the fifth, 25th, 50th, and 75th generations. The results showed that although the percentage of fluorescent cells decreased to 94.2% after 75 generations, the MFI of cells expressing EGFP exhibited minimal changes. The results indicated that the *H11* locus still maintained the transcriptional activity and expression stability of EGFP after culture for up to 2 and a half months. We randomly isolated 8 monoclonal cell strains CHO-CDbox-PAb-1∼8 from the CHO-CDbox-PAb cell pool for genome sequencing and proliferation curve comparison and discovered that the individual clones within the CHO-CDbox-PAb cell pool exhibited minor differences. Antibody secretion was analyzed across the first, 25th, and 50th generations. The results showed that the difference in antibody secretion between different monoclonal cells was less than 25%, with no more than 12% compared to the cell pool. When comparing the 50th-generation monoclonal to the first-generation antibody secretion, there was a decrease ranging from 5.27% to 12.98%. Additionally, the fluctuation of antibody secretion among various generations of each clone remained relatively constant, showing little disparity. This showed that there was a high degree of genetic and expression homogeneity among individual cells in the CHO-CDHE and CHO-CDbox-PAb cell pool, and it also proved that the *H11* locus could stably express antibodies after a long period of generation. However, the high expression level of the *H11* locus was not addressed in this study, and our laboratory will continue to compare the expression level differences between the *H11* locus and other commonly used hotspots horizontally.

Currently, researchers are interested in using multi-copy gene expression to enhance the production yields of antibodies in cells and have shown progress (Chusainow et al., 2010; Carver et al., 2020; Sergeeva et al., 2020; Srirangan et al., 2020). However, single-copy and multi-copy have their advantages, multi-copy gene expression can increase product yield to some extent, but single-copy gene expression is more controllable and traceable for a wide range of applications. For example, single-copy recombinase landing pads fulfill one of the present criteria for displaying antibodies in mammalian cells, as they enable the display of only one antibody per cell. The Flp-In™ cell line, currently the most popular for animal cell surface display, contains a stably integrated *FRT* site at the transcriptionally active genomic loci, ensuring the unique expression of the inserted gene (Zhou et al., 2010; Luo et al., 2018). However, after screening the cells for the target antibody in the station, it is time-consuming and laborious to extract the genome of the cells to obtain the corresponding gene sequences of the antibody and to reconstruct the antibody-expressing cells. Currently, cellular display systems for simultaneous display and secretion have also been developed to solve this problem. Insertion of the furin cleavage site gene upstream of the transmembrane protein genes resulted in the removal of most transmembrane proteins by furin at the Golgi apparatus (Zhou et al., 2013). Another mammalian expression system has been developed that simultaneous cell surface display and secretion of the same protein through alternate splicing of pre-mRNA (Horlick et al., 2013). These two approaches achieve synchronization of secretion and presentation at the protein and mRNA levels, respectively. The ratio of secretion to presentation is variable or random for each cell, and this can vary somewhat during the screening process after antibody presentation. With the CDbox system described in this paper, it was possible to transition from display to secretion at the gene level. Normally, after obtaining the display target antibody cells by screening, the Dre recombinase was used to delete all gene sequences including the *TM* gene in the middle of the two *roxP* sites to make the antibody secreted. Due to the single copy, the cells could only be in one of two states: displaying antibodies or secreting them. Through direct cell screening, monoclonal cell strains that secrete antibodies exclusively can also be obtained.

**FIGURE 7:**
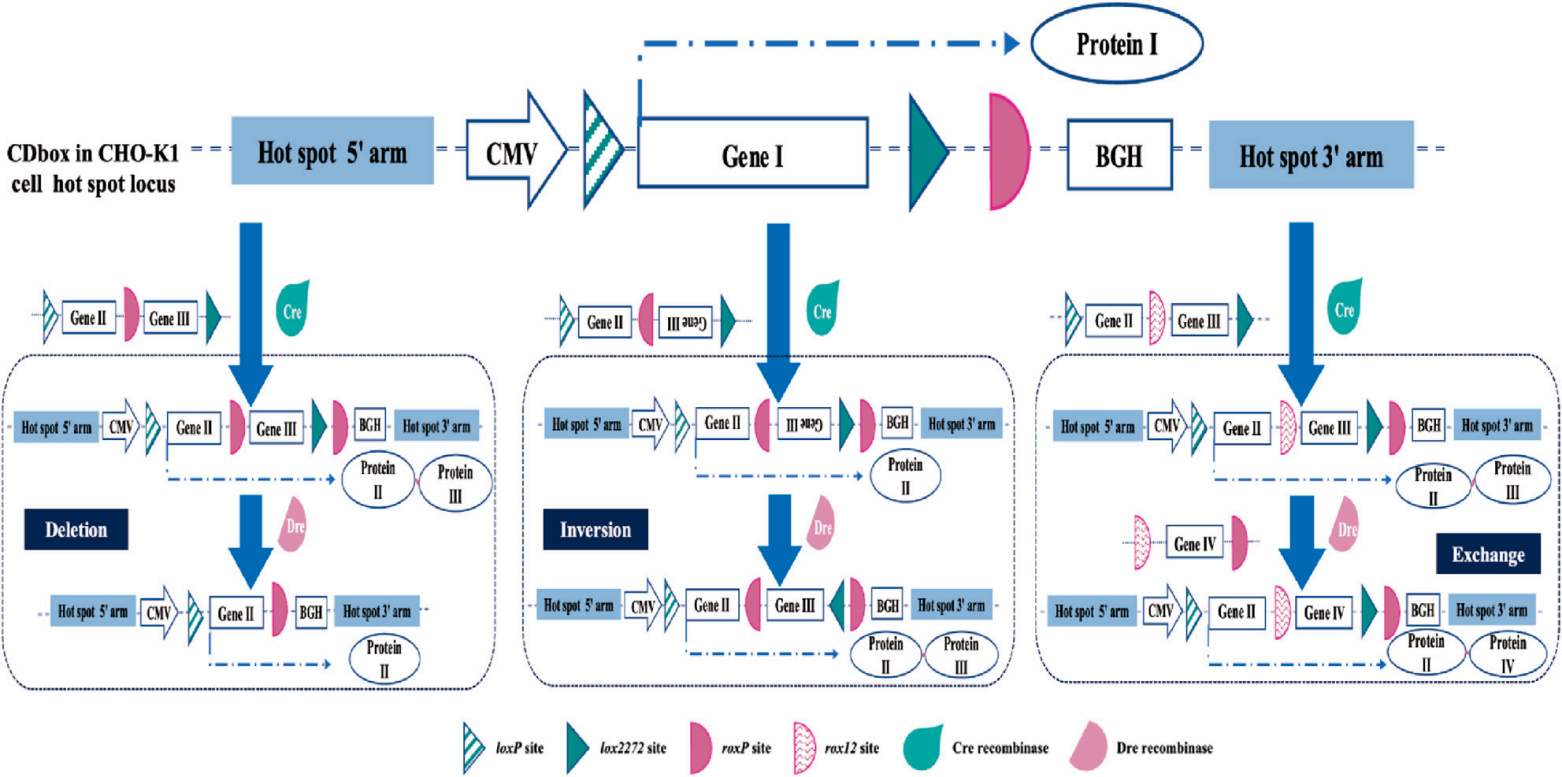
Extended application of the CDbox cell platform. In addition to the *H11* locus, CDbox can also be integrated and operated at any or multiple loci in the CHO genome. Based on the RMCE of the Cre/*lox* system, Dre/*rox* continues to realize deletion, inversion, and exchange of genes at this locus, supported by donor sequences.

We have developed a multifunctional expression cassette CDbox, which is not limited to the applications mentioned in this study. In addition to the *H11* locus, it can also be integrated and operated at any or multiple loci in the CHO genome to meet the different needs of CHO cell line research applications and cell line development. The CDbox expression cassette contains two Cre recombinase mutant sites, *loxP* and *lox2272,* which can undergo individual RMCE action for rapid insertion of the target gene at the target site. The presence of the Dre recombinase site does not interfere with the action of the Cre recombinase, and the donor can be constructed to perform different functions according to different needs. As shown in Figure 7, if the donor sequence contains the same orientation *roxP* site, it will perform the deletion, if it contains the reverse orientation *roxP* site, Dre recombinase will perform inversion, and if it contains another *roxP* variant site, such as *roxp12*, it will perform substitution.

In summary, we developed a CDbox expression cassette by Cre/*lox* and Dre/*rox* site-specific recombination systems and developed a versatile CHO-CDbox cell platform. This cell platform allows the rapid establishment of clones with stable exogenous gene expression and facilitates subsequent modification, thereby providing CHO cells with a valuable tool that has broad applications in protein production and gene function studies.

## Data Availability

The original contributions presented in the study are included in the article/Supplementary Material, further inquiries can be directed to the corresponding author.
