# Supplementary material for: Construction and application of a multifunctional CHO cell platform utilizing Cre/lox and Dre/rox site-specific recombination systems
Source: Front Bioeng Biotechnol. 2023 Dec 20;11:1320841. doi: 10.3389/fbioe.2023.1320841 (PMC10761530; doi:10.3389/fbioe.2023.1320841)
Supplement: Supplementary file 1 [file DataSheet1.docx]

Supplementary Material

# Supplementary Tables

**Supplementary Table 1: Synthesis of sgRNA oligonucleotides targeting *H11* locus.**

| sgRNA | Oligonucleotides Sequence (5**’**→3**’)** |
| --- | --- |
| F-*H11*-sgRNA1 | CACCGTATACACTTGAGCCAGTAGT |
| R-*H11*-sgRNA1 | AAACACTACTGGCTCAAGTGTATAC |
| F-*H11*-sgRNA2 | CACCGAGACCCTAGGCTGTAGACGT |
| R-*H11*-sgRNA2 | AAACACGTCTACAGCCTAGGGTCTC |
| F-*H11*-sgRNA3 | CACCGGGACGGTCATTAAGACTAC |
| R-*H11*-sgRNA3 | AAACGTAGTCTTAATGACCGTCCC |

**Supplementary Table 2: The PCR primer sequences used in the construction of the donor vector.**

| Gene Name | Primer  Name | Sequence (5**'**→3**')** | Underline |
| --- | --- | --- | --- |
| H11 5**'**arm | Fa | GGAAATTCCATATGCTCCCATACTGTGTCTACTCTC | NdeI |
|  | Ra | GGACTAGTGGATCCGAGCTCGGTACCACTGGCTCAAGTGTATACTT | BamHI-KpnI |
| H11 3**'**arm | Fb | GGTACCGAGCTCGGATCCACTAGTCCAGTGGGGGGAGGGGTGGTAAAGA | KpnI-BamHI |
|  | Rb | GCGGGGCCCAGTAATTGGATTACAGGTATGA | ApaI |
| mCherry | Fc | CCCAAGCTT*ATAACTTCGTATAGCATACATTATACGAAGTTAT*CGCCACCATGGTGAGCAAGGGCGAGGAG | HindIII-*loxP* |
|  | Rc | CAGACTTCCTCTGCCCTCCTTGTACAGCTCGTCCAT | T2A |
| T2A-PuroR | Fd | ATGGACGAGCTGTACAAGGAGGGCAGAGGAAGTCTG | Cherry |
|  | Rd | CCGCTCGAG*ATAACTTCGTATAAAGTATCCTATACGAAGTTAT*TCAGGCACCGGGCTTGCG | *lox2272*-XhoI |
| CDbox | Fe | CGGGGTACCGACATTGATTATTGACTAGTTATT | KpnI |
|  | Re | CGCGGATCCCCATAGAGCCCACCGCAT | BamHI |
| CDbox with H11 arm | Ff | CTCCCATACTGTGTCTACTCTC |  |
|  | Rf | AGTAATTGGATTACAGGTATGA |  |
| pcDNA3.1-*roxP* linear | Fr | CTCGAGA*TAACTTTAAATAATGCCAATTATTTAAAGTTA*TCTAGAGGGCCCGCGGTTC | XhoI-*roxP* |
|  | Rr | CCCAAGCTTAACTAGCCAGCTTGGGT | HindIII |
| *roxP*-EGFP | Fg | A*TAACTTTAAATAATGCCAATTATTTAAAGTTA*ATGGTGAGCAAGGGCGAGGAGCT | *roxP* |
|  | Rg | CCGCTCGAGCTACTTGTACAGCTCGTCCATGCCGAG | XhoI |
| HygR- *roxP* | Fh | CCGGAATTCGCCACCATGAAAAAGCCTGAACTCAC | EcoRI |
|  | Rh | *ATTAACTTTAAATAATTGGCATTATTTAAAGTTA*TTTCCTTTGCCCTCGGACGAGTGC | *Roxp* |
| HC-LC | Fi | CCGGAATTCGCCGCCATGGATTGGACATGGAG | EcoRI |
|  | Ri | TAACTTTAAATAATTGGCATTATTTAAAGTTATACACTCCCCGCGGTTGAAGGAC |  |
| HygR | Fj | ATGCCAATTATTTAAAGTTAGAGGGCAGAGGAAGTCTGCTAAC |  |
|  | Rj | CCGCTCGAGCTATTCCTTTGCCCTCGGACGAG | XhoI |
| VNAR | Fk | CCGGAATTCGCCGCC*ATGGATTGGACATGGAGAGTGTTTTGTCTGCTTGCGGTGACACCTGGC*GCACGTGTTGATCAGACCCCG | EcoRI-SP |
|  | Rk | TGGGCCATATTTGGATTCCTCGAGCGGATTAACCGTAACCGCCGTACCAT |  |
| Fc | Fl | CGGCGGTTACGGTTAATCCGCTCGAGGAATCCAAATATGGCCCACC |  |
|  | Rl | *CATTATTTAAAGTTA*TGGATCCCTTTCCCAGGCTCAGAGAGAGGGA | *roxP* |
| TM | Fm | GGATCCA*TAACTTTAAATAATGCCAATTATTTAAAGTTA*GCTGTGGGCCAGGACACGC | *roxP* |
|  | Rm | TTCCTCTGCCCTCTGCACTGCCACGTGGCTTCTTCTGCCAAAG |  |
| T2A-HygR | Fn | CTTTGGCAGAAGAAGCCACGTGGCAGTGCAGAGGGCAGAGGAAG | T2A |
|  | Rn | CCGCTCGAGCTATTCCTTTGCCCTCGGACG | XhoI |

**Supplementary Table 3: The primer sequences used for** **5'/3' Junction PCR.**

| Primer | Oligonucleotides Sequence (5**'**→3**')** |
| --- | --- |
| F_out_ | TGACTGGTTCTGTGCTTATTTCTTC |
| R_in_ | CTTCAGCTTGGCGGTCTGGGTGC |
| F_in_ | GCAACCTCCCCTTCTACGAGC |
| R_out_ | GCCAGAAGAGGGTTTTGAATGTC |
| F_inHL_ | ATGCGATCGCTGCGGCCGATCTTA |
| R_inHL_ | ACAAAACACTCTCCATGTCCAATCCA |

# Supplementary Figures

#
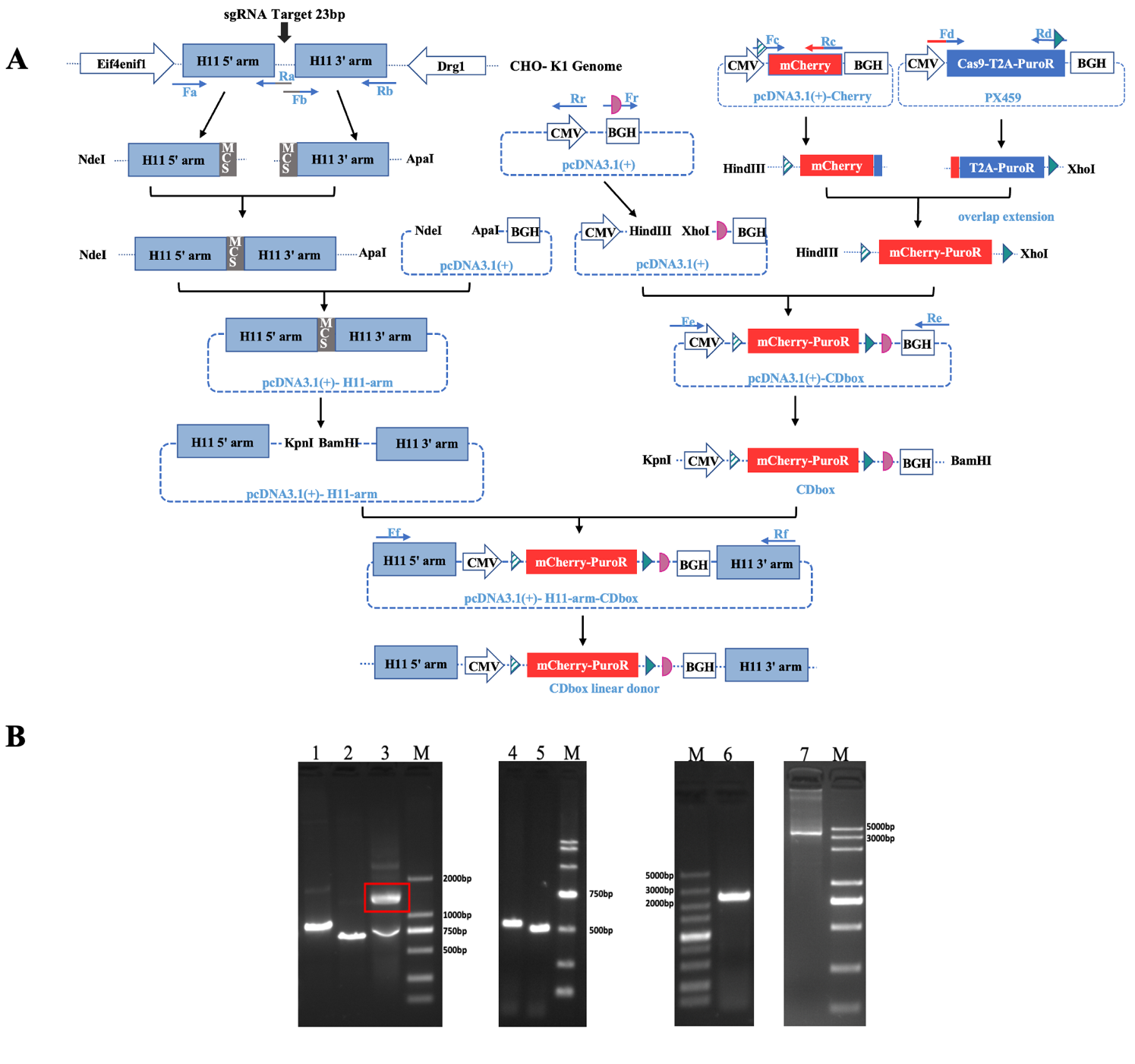


# Supplementary Figure 1. Schematic diagram of the process of constructing CDbox liner donor. (A) CDbox donor component sources and constructions. (B) PCR amplification of different gene elements of the CDbox donor. Lane 1, T2A-PuroR-*lox2272-*XhoI; lane 2, HindIII-loxP-mCherry; lane 3, HindIII-*loxP*-mCherry-PuroR-lox2272-XhoI; lane 4, sgRNA1 5' arm; lane 5, sgRNA1 3' arm; lane 6, KpnI-CMV-*loxP*-mCherry-PuroR-*lox2272*-*roxP*-BGH-BamHI; lane 7, 5' arm-CMV-*loxP*-mCherry-PuroR-*lox2272*-*roxP*-BGH -3' arm.


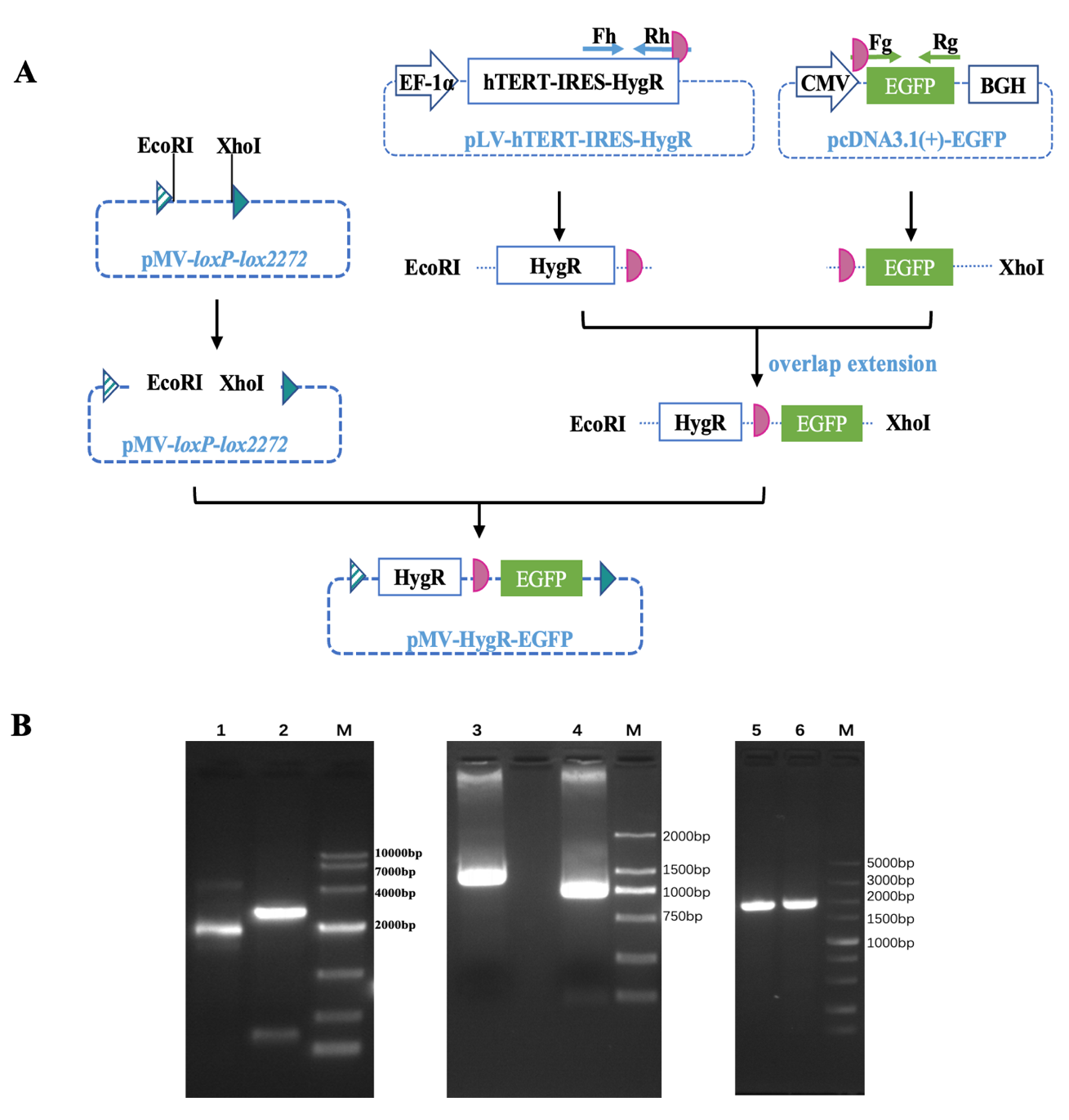


**Supplementary Figure 2. Schematic diagram of the process of constructing** **pMV-HygR-EGFP** **donor** **vector.** (A) pMV-HygR-EGFP donor vector component sources and constructions. (B) PCR amplification of different gene elements of the HygR-EGFP donor. Lane 1, pMV-*loxP*-*lox2272* plasmid; lane 2, pMV-*loxP*-*lox2272* plasmid digested by EcoRI and XhoI; lane 3, EcoRI-HygR-*roxP*; lane 4, *roxP*-EGFP-XhoI; lane 5 and lane 6, EcoRI-HygR-*roxP*-EGFP-XhoI;


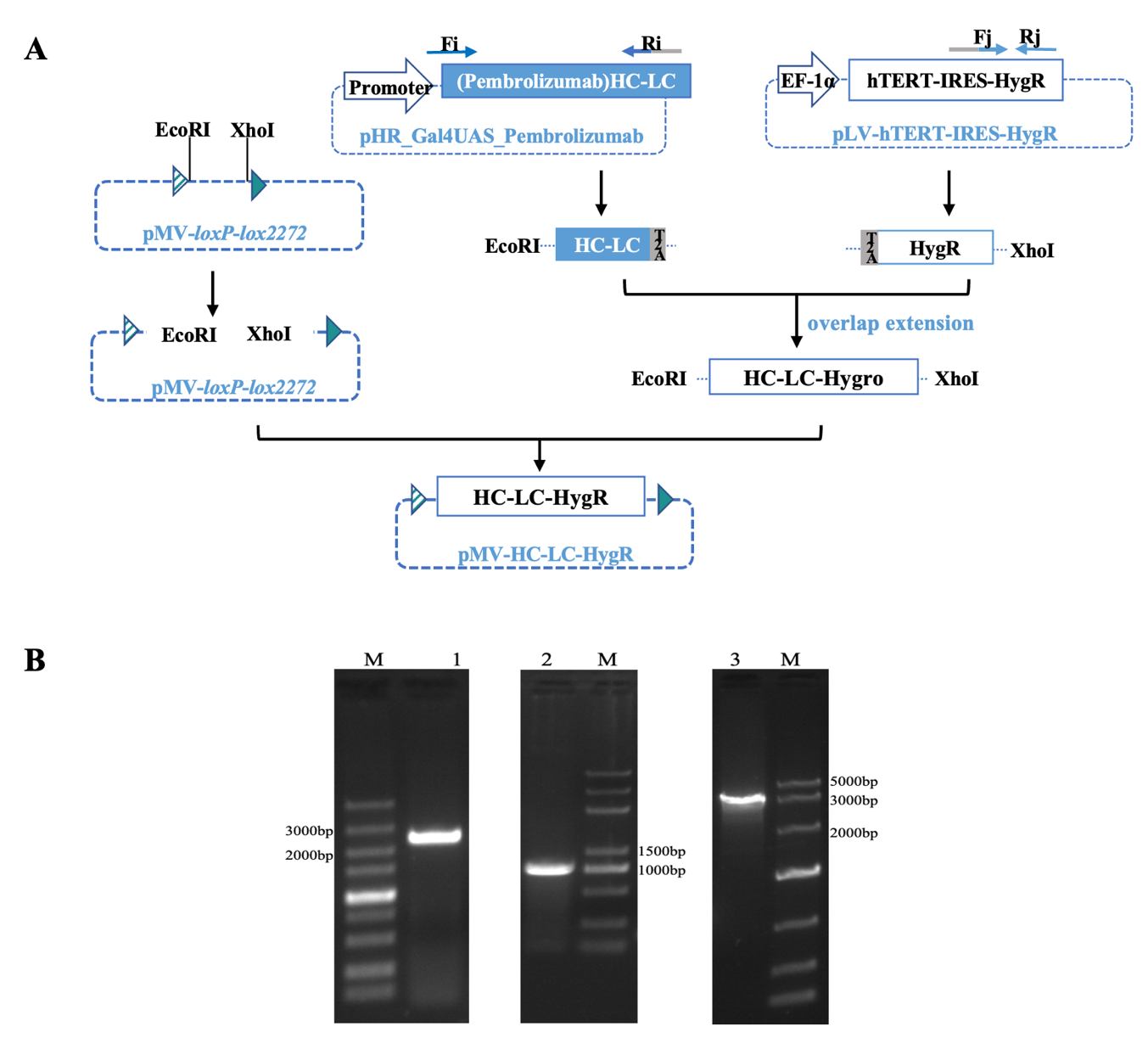


**Supplementary Figure 3. Schematic diagram of the process of constructing** **pMV-HC-LC-HygR donor vector.** (A) pMV-HC-LC-HygR donor vector component sources and constructions. (B) PCR amplification of different gene elements of HC-LC-HygR donor. Lane1, EcoRI- HC-LC-T2A; lane 2, T2A-HygR-XhoI; lane 3, EcoRI-HC-LC-HygR-XhoI;


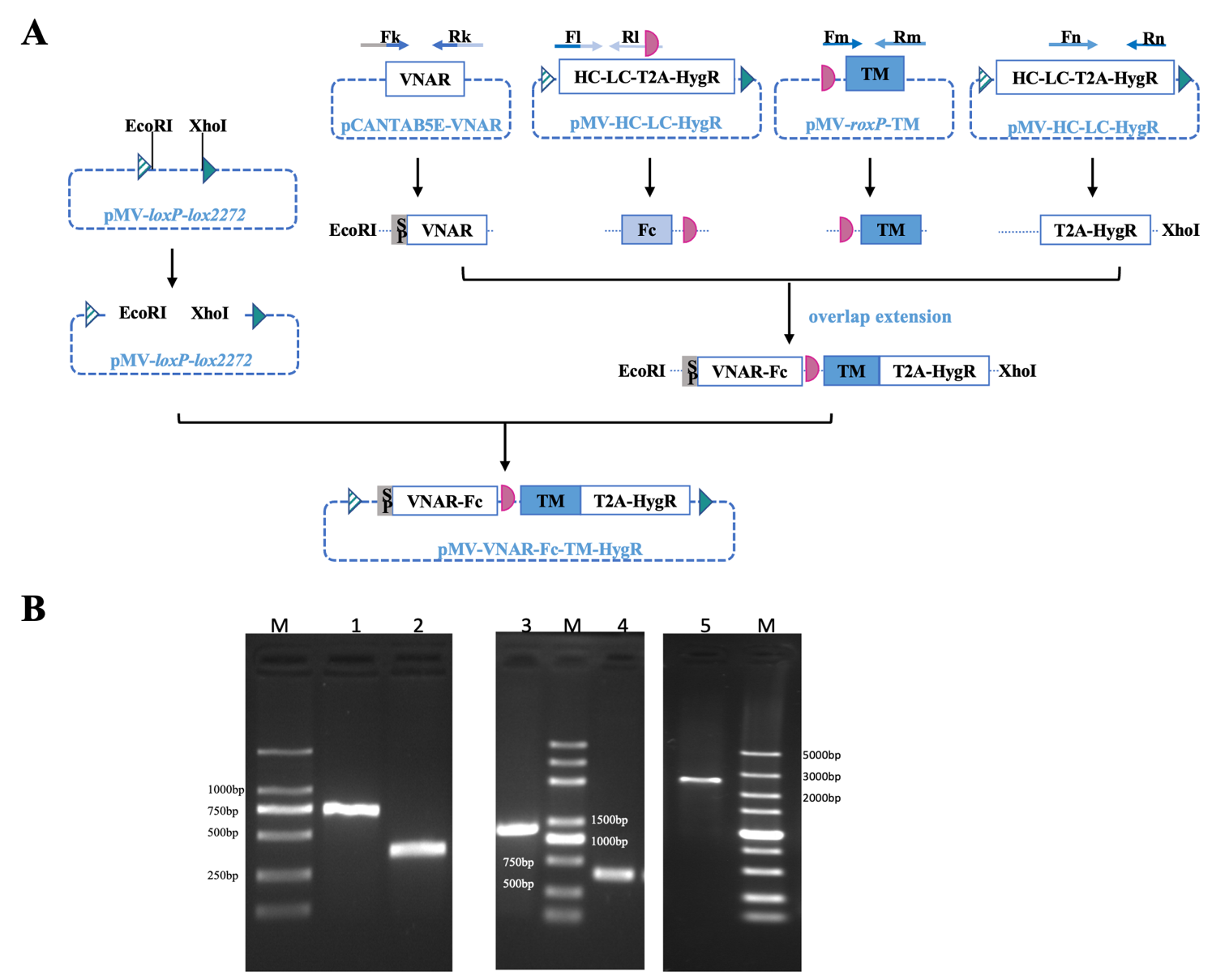


**Supplementary Figure 4. Schematic diagram of the process of constructing pMV-****VNAR-Fc-TM-HygR donor vector.** (A) pMV-VNAR-Fc-TM-HygR donor vector component sources and constructions. (B) PCR amplification of different gene elements of pMV-VNAR-Fc-TM-HygR. Lane1, Fc-*roxP*; lane 2, *roxP*-TM; lane 3, T2A-HygR-XhoI; lane 4, EcoRI-SP-VNAR; lane 5, EcoRI-VNAR-Fc-TM-HygR-XhoI;


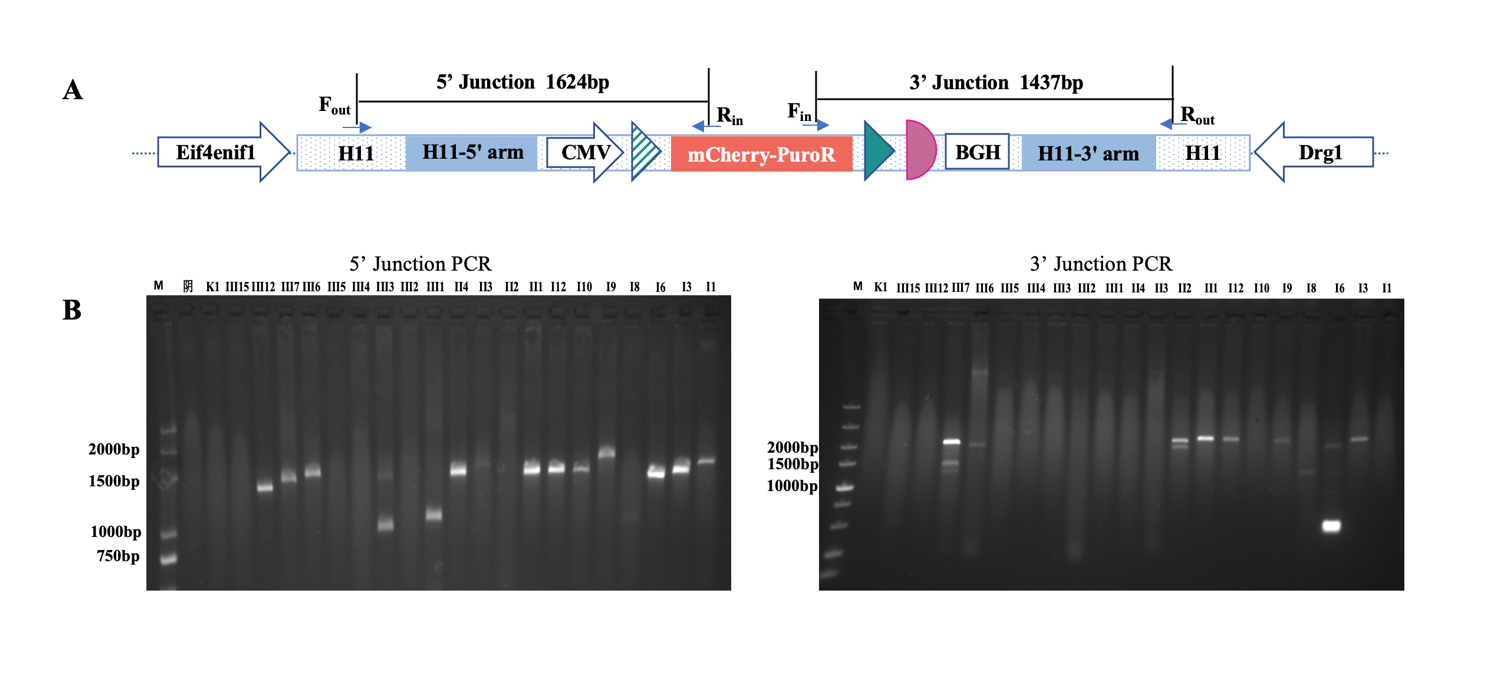


**Supplementary Figure 5.** **Junction PCR identification of positive monoclonal cell strains.** (A) The primer position and product fragment size of 5'/3' junction PCR. (B) Agarose gel results of 5'/3' Junction PCR.


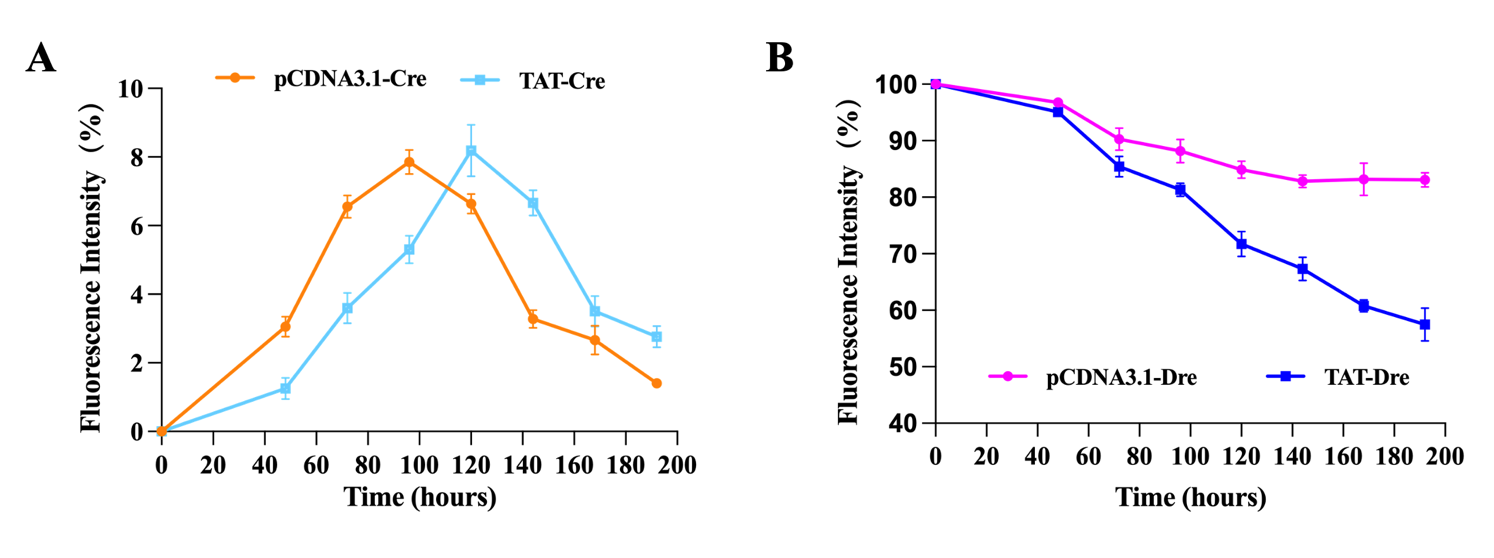


**Supplementary Figure 6. Efficiency of different forms of Cre and Dre recombinase in CHO cells.** (A) Comparison of the efficiency of different forms of Cre recombinase acting on cellular genomes. Cre recombinase entered CHO cells in the form of plasmids or transmembrane peptides (mean ± S.D., n = 2 independent experiments). (B) Comparison of the efficiency of different forms of Dre recombinase acting on cellular genomes. Dre recombinase entered CHO cells in the form of plasmids or transmembrane peptides (mean ± S.D., n = 2 independent experiments).

**
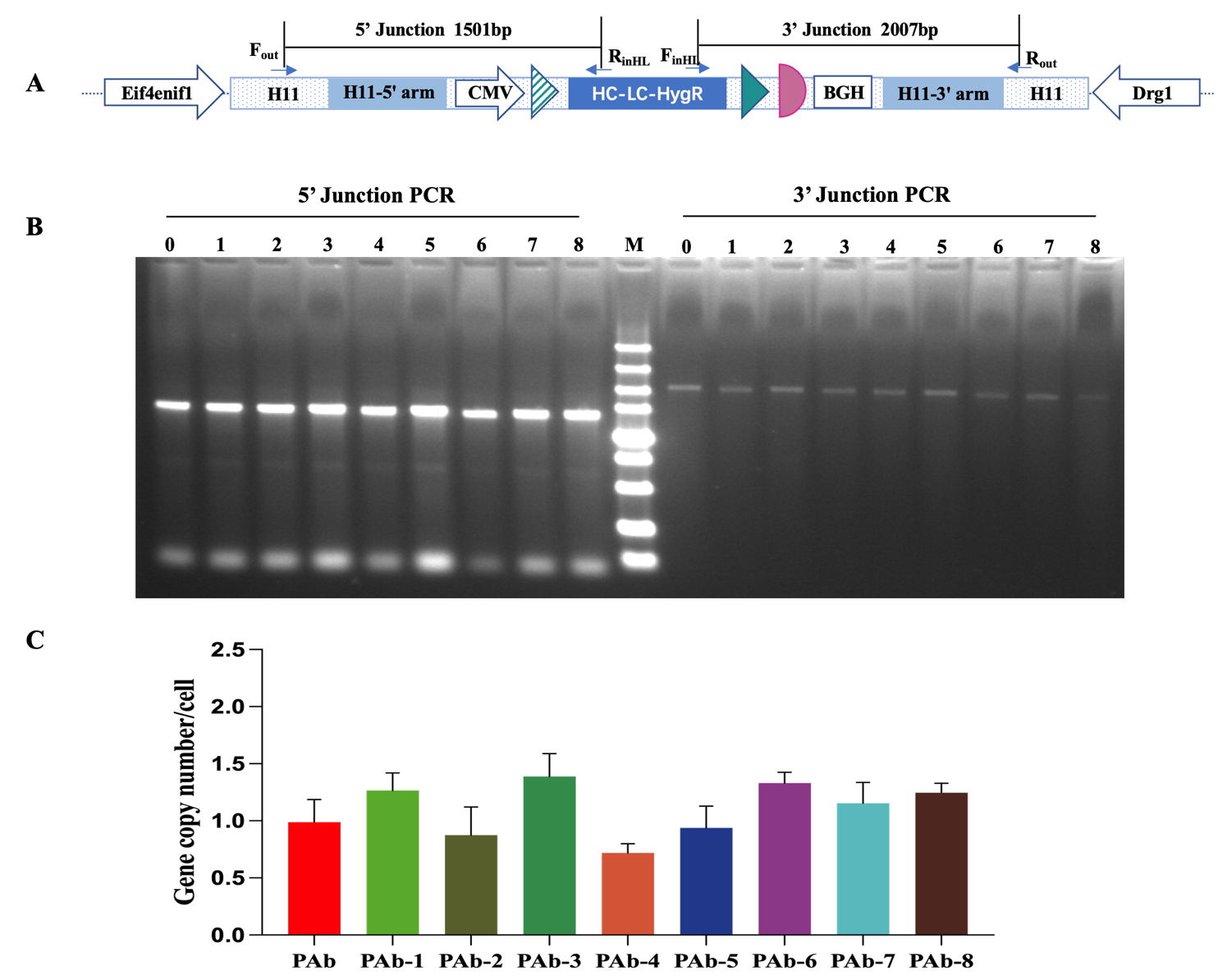
**

**Supplementary Figure 7. Junction PCR identification of** **CHO-CDbox-PAb cell subcones.** (A) The primer position and product fragment size of 5'/3' junction PCR. (B) Agarose gel results of 5'/3' Junction PCR. (C) Pembrolizumab antibody gene copy number in the CHO-CDbox-PAb cells pool and its monoclonal cell line (mean ± S.D., n = 3 independent experiments).


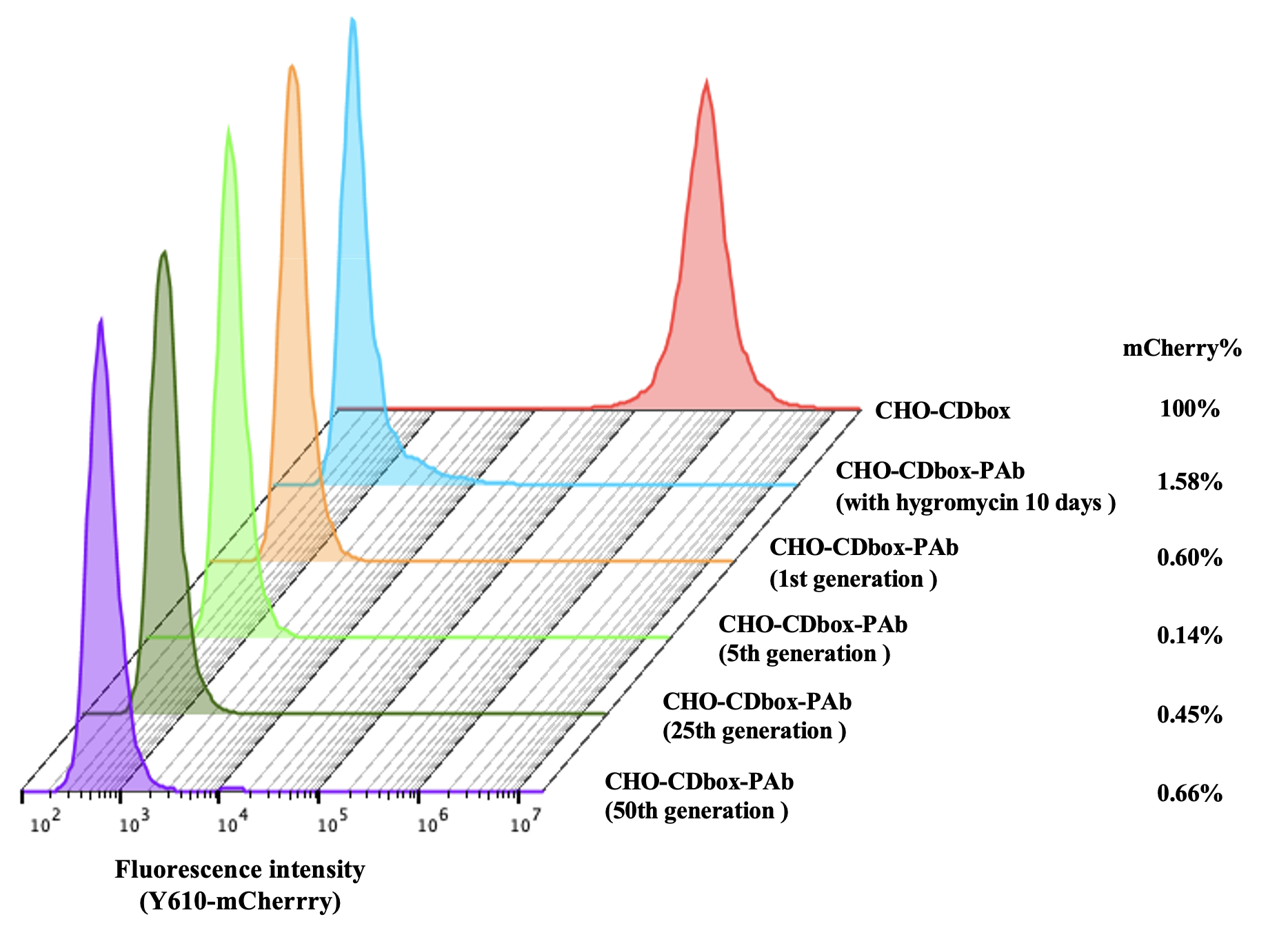


**Supplementary Figure 8.** The mCherry fluorescence expression rate of CHO-CDbox-PAb cells pool at various time points. The fluorescence rate was detected by flow cytometry after 10 days of hygromycin screening and in the 1st, 5th, 25th, and 50th generations using CHO-CDbox cells as the experimental control.
